# ALOMYbase, a resource to investigate non-target-site-based resistance to herbicides inhibiting acetolactate-synthase (ALS) in the major grass weed *Alopecurus myosuroides* (black-grass)

**DOI:** 10.1186/s12864-015-1804-x

**Published:** 2015-08-12

**Authors:** Jeanne Aude Christiane Gardin, Jérôme Gouzy, Sébastien Carrère, Christophe Délye

**Affiliations:** INRA, UMR1347 Agroécologie, 17 rue de Sully, F-21000 Dijon, France; INRA, UMR441 LIPM, F-31326 Castanet-Tolosan, France

**Keywords:** RNA sequencing, Transcriptomics, Herbicide response, Non-target-site resistance, *Alopecurus myosuroides* (black-grass), Weed, Acetolactate synthase, Acetohydroxyacid synthase

## Abstract

**Background:**

Herbicide resistance in agrestal weeds is a global problem threatening food security. Non-target-site resistance (NTSR) endowed by mechanisms neutralising the herbicide or compensating for its action is considered the most agronomically noxious type of resistance. Contrary to target-site resistance, NTSR mechanisms are far from being fully elucidated. A part of weed response to herbicide stress, NTSR is considered to be largely driven by gene regulation. Our purpose was to establish a transcriptome resource allowing investigation of the transcriptomic bases of NTSR in the major grass weed *Alopecurus myosuroides* L. (*Poaceae*) for which almost no genomic or transcriptomic data was available.

**Results:**

RNA-Seq was performed from plants in one F2 population that were sensitive or expressing NTSR to herbicides inhibiting acetolactate-synthase. Cloned plants were sampled over seven time-points ranging from before until 73 h after herbicide application. Assembly of over 159M high-quality Illumina reads generated a transcriptomic resource (ALOMYbase) containing 65,558 potentially active contigs (N50 = 1240 nucleotides) predicted to encode 32,138 peptides with 74 % GO annotation, of which 2017 were assigned to protein families presumably involved in NTSR. Comparison with the fully sequenced grass genomes indicated good coverage and correct representation of *A. myosuroides* transcriptome in ALOMYbase. The part of the herbicide transcriptomic response common to the resistant and the sensitive plants was consistent with the expected effects of acetolactate-synthase inhibition, with striking similarities observed with published *Arabidopsis thaliana* data. *A. myosuroides* plants with NTSR were first affected by herbicide action like sensitive plants, but ultimately overcame it. Analysis of differences in transcriptomic herbicide response between resistant and sensitive plants did not allow identification of processes directly explaining NTSR. Five contigs associated to NTSR in the F2 population studied were tentatively identified. They were predicted to encode three cytochromes P450 (CYP71A, CYP71B and CYP81D), one peroxidase and one disease resistance protein.

**Conclusions:**

Our data confirmed that gene regulation is at the root of herbicide response and of NTSR. ALOMYbase proved to be a relevant resource to support NTSR transcriptomic studies, and constitutes a valuable tool for future research aiming at elucidating gene regulations involved in NTSR in *A. myosuroides*.

**Electronic supplementary material:**

The online version of this article (doi:10.1186/s12864-015-1804-x) contains supplementary material, which is available to authorized users.

## Background

Agrestal weeds are the major biotic cause for crop yield losses [1]. Most weeds are annual or short-lived wild plant species. They thrive in agricultural ecosystems because they have evolved traits enabling them to withstand crop competition and cultural practices, including herbicide applications, aimed at disrupting their demography [2, 3]. Understanding weed success requires unravelling the genetic basis of these traits, a task far from being achieved today [3]. Prominent among those traits is resistance to herbicides that has now evolved in 246 weed species [4] in response to the powerful and recurrent selective pressure exerted by herbicide applications [5]. The evolution of herbicide resistance in weed populations can ultimately result in the disruption of herbicide efficacy, leading to crop failure [6].

Basically, mechanisms of resistance to herbicides can be categorised into two classes according to their genetic control [5]. Monogenic resistance is governed by allele(s) of a single gene, while polygenic resistance is governed by allele(s) of a set of genes, with “allele” meaning a variant of a wild-type gene displaying differences in its protein-coding sequence and/or its regulatory region [5]. Target-site-based resistance endowed by mutations at the gene encoding the herbicide target protein is an example of monogenic resistance that is now well elucidated in weeds [5, 6]. Non-target-site based resistance (NTSR) endowed by mechanisms neutralising the herbicide or compensating for its action is most often a case of polygenic resistance [5, 7, 8]. NTSR can confer resistance to herbicides with different modes of action and is considered the most agronomically noxious type of herbicide resistance [5, 6]. NTSR is overall the most widespread and frequent type of resistance in grass weeds [5, 6]. The literature available suggests that NTSR mechanisms are part of the pathways involved in the response of weed plants to the herbicide stress. Accordingly, NTSR is considered to be largely driven by inheritable differences in the expression patterns of one or more genes between resistant and sensitive plants [9, 10]. These differences can be constitutive and/or induced by herbicide application [9, 10]. Cytochromes P450, glutathione-S-transferases, glycosyltransferases, esterases, ABC transporters and/or peroxidases have been shown to play a major role in herbicide response and in NTSR (reviewed in [9, 10]). While a few NTSR genes belonging to these families have recently been identified [11–19], the majority of the genetic mechanisms underlying NTSR remain to be elucidated [10].

Elucidating the genetic basis of NTSR requires being able to unravel the genetic bases of herbicide stress response in weeds, and to identify genetic differences between resistant and sensitive plants before and after herbicide application [9, 10]. This is now feasible thanks to the tremendous development of the Next-Generation Sequencing technologies (reviewed in [20]) that enable establishment of transcriptomic resources for plant species without the need for associated genomic resources [21]. Next generation sequencing technologies allow comprehensive transcriptome sequencing (RNA-sequencing or RNA-Seq) that produces both qualitative data (transcript sequences) and quantitative data (transcript expression level) with an unprecedented level of sensitivity and accuracy [22–24]. Accordingly, RNA-Seq is considered a highly promising way of unravelling the genetic control of complex traits in weeds [3, 25]. Yet, despite the acknowledgement of the potential of transcriptome-wide sequencing to study weed response to herbicides and NTSR [10], only a few studies have implemented this approach to date [15–19, 26].

*Alopecurus myosuroides* L. (black-grass) is a diploid grass (*Poaceae*) weed with no associated genomic or transcriptomic resources. *A. myosuroides* is a major weed of winter crops in North-Western Europe that can be responsible for substantial yield losses [27]. *A. myosuroides* has evolved resistance to six herbicide modes of action [4], including leaf-applied herbicides that are the herbicides most used to control this species. In *A. myosuroides*, resistance to leaf-applied herbicides is mostly due to polygenic NTSR mechanisms [5, 28, 29]. The major group of leaf-applied herbicides used against *A. myosuroides* are acetolactate synthase (ALS) inhibitors. ALS is a key enzyme in the branched-chain amino acids (BCAAs) biosynthesis pathway [30, 31]. Physiological effects of ALS inhibitors on the cellular amino-acid pools, protein turnover and carbohydrate metabolism have been described in crop or model species (reviewed in [30]). To the best of our knowledge, the transcriptomic response of a plant to the stress caused by an ALS inhibitor had so far only been investigated in a few studies considering model or crop species [32–35].

Our purpose was to establish the first transcriptome resource for *A. myosuroides* (ALOMYbase) using Illumina sequence data obtained from a time-course experiment. To check the relevance of ALOMYbase for the identification of genetic determinants of NTSR to ALS inhibitors, the transcriptomic response of *A. myosuroides* plants resistant or sensitive to an ALS inhibitor was investigated. Five contigs potentially involved in NTSR to ALS inhibitors were identified as an additional outcome of this study.

## Results

### Establishing ALOMYbase: *A. myosuroides* transcriptome sequencing and data assembly

The expected phenotypes of all plants used for RNA-Seq (i.e., three resistant and three sensitive F2 plants) were confirmed by rating the corresponding phenotype control clones 4 weeks after herbicide application. Raw 100-base single-end sequence reads were subjected to quality control using Phred scaled quality score. Overall, 92.4 % of the sequenced bases had a quality score ≥ 30 and 95.7 % of the sequence reads passed quality filters and matched Illumina’s quality requirements. The proportion of undetermined bases was 0.009 and 0.001 % in the two flow cells used for sequencing. After filtering out low quality reads, homopolymers and short reads, 159,089,080 100-base single-end reads were generated from the 14 cDNA libraries. The number of reads was similar in the different libraries (Additional file 1: Figure S1).

**Fig. 1:**
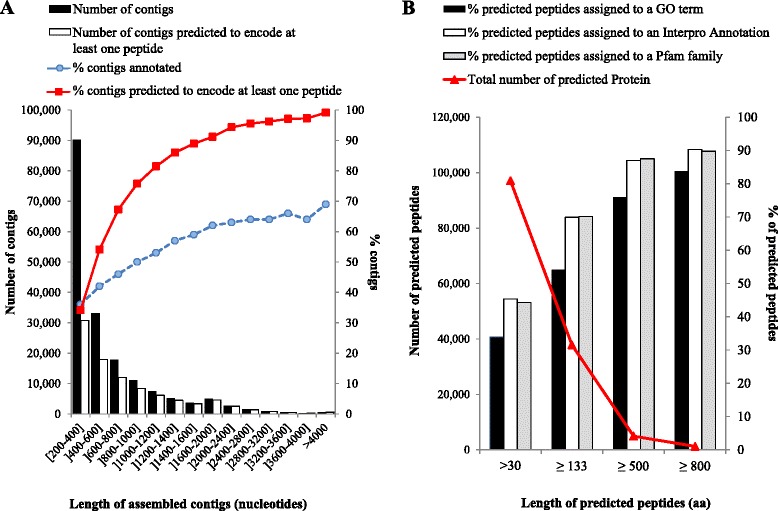
ALOMYbase peptide contents and annotation. **a** Number of assembled contigs and number of contigs predicted to encode at least one peptide according to the contig size range (left axis), and percentage of contigs predicted to encode at least one peptide and percentage of contigs successfully assigned an InterPro annotation (right axis). **b** ALOMYbase predicted peptide contents and peptide annotation

*De novo* assembly of the 100-base reads yielded 180,117 unique sequences ranging from 200 to 17,734 nucleotides in length (Table 1). The length distribution of the assembled contigs is shown in Fig. 1a. The proportion of contigs with at least one mapped read was similar in all experimental modalities, except the resistant pool at 48 h After Treatment (HAT) where only 50 % of the contigs assembled had at least one mapped read (Additional file 1: Figure S2). This experimental modality was thus considered dubious, and the whole 48HAT time-point was removed from further analyses.

**Table 1:** ALOMYbase statistics

ALOMYbase – *A. myosuroides* transcriptome resource	
Total number of reads	159,089,080
Total assembled contigs	180,117
Total size of the assembly	110.87 Mb
Average contig size	616 nucleotides
N50	835 nucleotides
Total contigs after filtering^a^	65,558
Total size of the assembly after filtering^a^	68.59 Mb
Average contig size after filtering^a^	1046
N50 after filtering^a^	1240
Total predicted peptides after filtering^b^	32,138
% predicted peptides with a GO annotation^b^	56.9
% predicted peptides with an Interpro annotation^b^	74.3

^a^Filters: contig size ≥ 400 nucleotides, RPKM ≥ 1.8 in at least one experimental modality

^b^Filers: contig size ≥ 400 nucleotides, RPKM ≥ 1.8 in at least one experimental modality, and peptide length > 134 amino-acids

### Functional annotation and contig expression data

Automated search for coding sequences in all the assembled contigs using FrameDP identified 97,079 predicted peptides with an average length of 166 amino-acids. Peptide size ranged from 30 to 4135 amino-acids. Overall, 33.9, 45.3 and 44.3 % of the predicted peptides could be assigned a GO term, an InterPro domain or a Pfam family, respectively (Fig. 1b). Small contigs and predicted peptides of small size that probably corresponded to truncated contigs were poorly annotated (Fig. 1b). Thus, assembled contigs shorter than 400 nucleotides or encoding predicted peptides shorter than 134 amino-acids were considered as assembly waste and discarded.

RPKM counts were computed in each experimental modality for every contig. Contigs with low RPKM values could be assembly artefacts. As proposed before [36–38], a contig was considered potentially expressed if it had an average 2-fold sequencing coverage. As the reads used for *de novo A. myosuroides* transcriptomic data assembly were 100-base long, this corresponded to a RPKM value of 1.8. Accordingly, only contigs with a RPKM count ≥1.8 in at least one experimental modality were considered expressed. All the other contigs were discarded. Of the 90,036 assembled contigs with a length ≥400 nucleotides, 65,558 that had a RPKM value ≥ 1.8 in at least one library were considered potentially from expressed genes and used for subsequent analyses. These contigs had a N50 value of 1240 nucleotides and were predicted to encode 32,138 peptides with a length > 134 amino-acids, of which 56.9 and 74.0 % could be assigned a GO term and/or an InterPro domain, respectively (Table 1). In the following, the name “ALOMYbase” will refer to the 65,558 contigs and the corresponding 32,138 predicted peptides sequences.

**Fig. 2:**
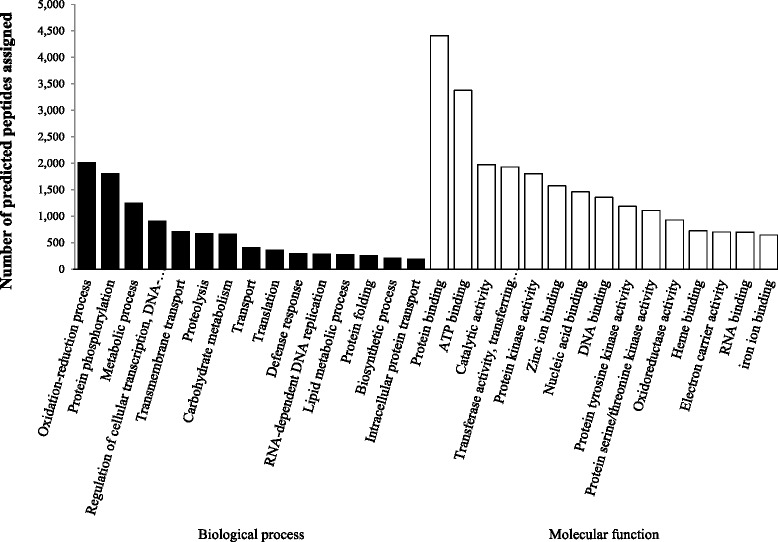
ALOMYbase top 15 GO terms in the categories Biological Process (black bars) and Molecular Function (white bars)

The 15 GO terms in the Molecular Function and Biological Process categories most represented in ALOMYbase are shown in Fig. 2. Terms related to oxidation-reduction process, protein phosphorylation and metabolic process contained the highest number of predicted peptides in the Biological Process category. “Protein binding” and “ATP binding” contained the highest number of predicted peptides in the Molecular Function category (Fig. 2).

### Comparison of the predicted peptide contents of ALOMYbase with other grass species

The 32,138 predicted peptides in ALOMYbase were compared to those encoded in the genomes of all grass (*Poaceae*) species with a fully sequenced genome, i.e., *B. distachyon*, *H. vulgare*, *Z. mays*, *O. sativa* and *S. bicolor* that contained 30,994, 79,379, 63,532, 49,059 and 29,448 predicted peptides, respectively. A total of 30,443 OrthoMCL groups were identified among the five species and ALOMYbase. Of these, 13,845 (45.5 %) were found in ALOMYbase, while 17,556 (57.7 %) to 20,892 (68.6 %) were found in any of the individual species (Additional file 2: Figure S3). Overall, 9328 OrthoMCL groups (30.6 %) were present both in ALOMYbase and in all the five grass genomes. The highest number of OrthoMCL groups exclusively shared by ALOMYbase and one of the grass genomes was observed for *H. vulgare* and *B. distachyon* (511 and 217 groups, respectively) (Additional file 2: Figure S3). A total of 833 OrthoMCL groups were specific to ALOMYbase. Based on their Interpro annotation, they mostly contained putative cytochromes P450 and regulatory proteins.

A total of 4062 Pfam families were identified among the five grass genomes and ALOMYbase. Among these, 3374 were identified in ALOMYbase, while 3658 to 3758 were identified in the five grass genomes. Considering the number of predicted peptides assigned to each Pfam family, the 22 Pfam families most represented in ALOMYbase had a similar ranking in all grass genomes (Additional file 3: Table S1). Three of these families were potentially involved in herbicide response: cytochrome P450, UDP-glycosyltransferase and ABC transporter. Overall, considering the 4062 Pfam families, Pearson correlations of the rankings of Pfam families were 0.51, 0.47, 0.45, 0.44 or 0.42 between ALOMYbase and *B. distachyon*, *S. bicolor*, *O. sativa*, *Z. mays* or *H. vulgare* genomes, respectively.

**Fig. 3:**
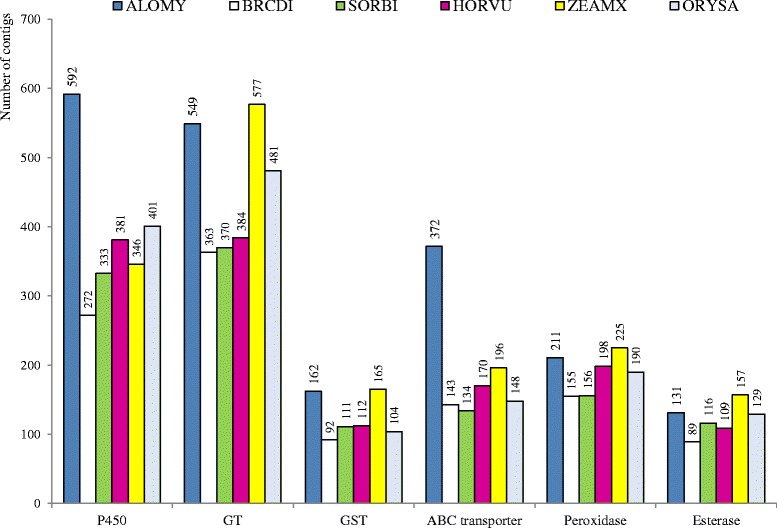
Peptides assigned to six Pfam families potentially involved in herbicide response and/or in NTSR. The number of peptides in these families is given for ALOMYbase and the five fully sequenced grass genomes. P450, cytochromes P450; GT, glycosyltransferases; GST, glutathione-S-transferases; ALOMY, ALOMYbase; BRCDI, *B. distachyon*, SORBI, *S. bicolor*; HORVU, *H. vulgare*; ZEAMX, *Z. mays*; ORYSA, *O. sativa*

Considering Pfam families potentially involved in herbicide response and/or in NTSR, ALOMYbase contained 592, 549, 162, 372, 211 and 131 putative peptides assigned to cytochromes P450, glycosyltransferases, glutathione-S-transferases, ABC transporters, peroxidases or esterases, respectively (Fig. 3). The number of putative peptides assigned to these Pfam families were roughly similar in ALOMYbase and in the five grass genomes, except cytochromes P450 or ABC transporters that were particularly numerous in ALOMYbase.

The comprehensiveness of ALOMYbase was assessed using CEGMA. CEGMA checks the presence in genome or transcriptome assemblies of a set of 248 proteins from housekeeping genes considered widely conserved among eukaryotes [39]. CEGMA analysis of ALOMYbase identified 152 of the 248 eukaryotic core proteins (61.3 %) as “complete”, “complete” being defined as >70 % alignment length with a core protein. As a comparison, CEGMA analysis of the transcript sets available for each of the five grass genomes identified 242 proteins (97.6 %) in *B. distachyon*, 246 proteins (99.2 %) in *S. bicolor*, 237 (95.6 %) in *O. sativa*, 233 (93.9 %) in *Z. mays* and 198 (79.8 %) in *H. vulgare* as complete.

### RT-qPCR validation of ALOMYbase expression data

RNA-Seq-based contig expression data were validated by measuring the expression of 21 contigs over the time-course using RT-qPCR. These contigs were randomly selected. Two had a stable RNA-Seq expression pattern and 19 had a RNA-Seq expression pattern varying over time or between phenotypes (Additional file 3: Table S2). The expression level of the 21 contigs in the 42 individual samples showed substantial variation among experimental modalities and among individual plants within modalities. Variation was also observed among plants with the same phenotype in the same experimental modality (Additional file 4: Figure S4). Despite this variation, the average contig expression levels computed for the three plants in the resistant pool and for the three plants in the sensitive pool at each time-point matched the corresponding RNA-Seq expression data (Pearson’s correlation coefficient value = 0.82 for the 21 contigs) (Additional file 5: Figure S5).

### Transcriptomic response to the herbicide application

**Fig. 4:**
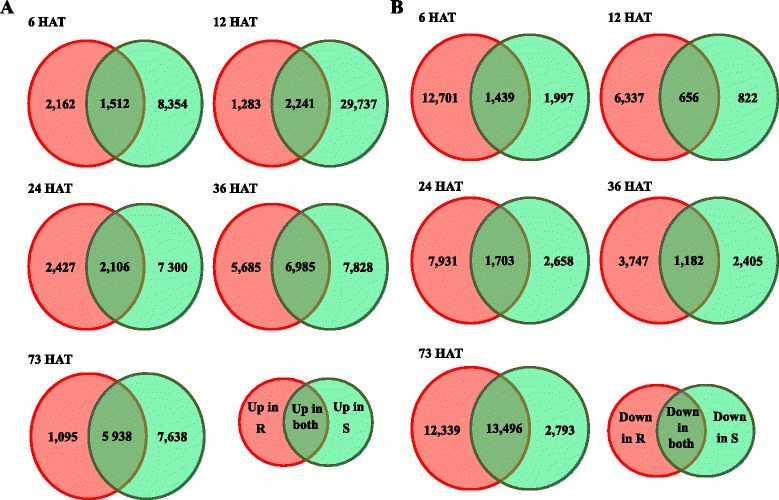
Number of contigs regulated by herbicide application. Venn diagrams show the number of contigs up-regulated (**a**) or down-regulated (**b**) in the resistant pool (R), in the sensitive pool (S) or in both phenotypes after herbicide treatment. Each herbicide-treated modality was compared to the UT of the corresponding phenotype. xHAT, x hours after herbicide application

The Untreated (UT), 6HAT, 12HAT, 24HAT, 36HAT and 48HAT time-points originated from the same time-course experiment. The 73HAT time-point originated from a different time course experiment. At 73HAT, 14,671 contigs were up-regulated compared to UT and 28,628 contigs were down-regulated compared to UT (Fig. 4). These numbers were not different from those at the five other time-points after herbicide application (19,405 and 10,894 on average in the other time-points after herbicide application; outlier test based on the assumption of normal distribution of data at a significance level of 0.05). Furthermore, the RPKM values computed for the three reference genes used for RT-qPCR data normalization at 73HAT in the resistant pool and in the sensitive pool were not different from those in the other 12 experimental modalities (outlier test based on the assumption of normal distribution of data at a significance level of 0.05). Including the 73HAT time-point in the subsequent analyses was thus deemed relevant.

A total of 57,427 contigs (87.6 % of the contigs in ALOMYbase) were up- or down-regulated in the resistant and/or in the sensitive pool in at least one herbicide-treated modality compared to the respective UT modalities. Among these, 41,843 contigs were up-regulated and 39,626 contigs were down-regulated in the resistant and/or in the sensitive pool in at least one herbicide-treated modality compared to the respective UT experimental modalities.

### Investigating the transferability of *Arabidopsis thaliana* transcriptional markers for the response to ALS inhibitors to *A. myosuroides*

A previous study investigating *Arabidopsis thaliana* transcriptome-wide response to four herbicides inhibiting ALS identified a set of 101 genes which expression pattern was linked to the response to these herbicides in *A. thaliana* and *Brassica napus* [34]. In both species, a subset of 46 genes had a similar regulation pattern in response to four ALS inhibitors (“group 1 markers”), while the regulation pattern of the remaining 55 genes varied with the herbicide (“group 2 markers”) [34]. To investigate whether this transcriptional signature was conserved in *A. myosuroides*, identification of putative homologues to the 101 *A. thaliana* genes in ALOMYbase was conducted using BLASTp. For each *A. thaliana* gene, the predicted peptide in ALOMYbase with the best BLASTp hit (i.e. the smallest E-value) was retained and considered the most likely homolog. Only predicted peptides with a significant homology (E-value < 10^−5^) were considered (Additional file 3: Table S3). Two *A. thaliana* genes (AT5G61020 and AT1G55500) shared the highest homology with the same predicted peptide in ALOMYbase, and seven had no significant homolog in ALOMYbase. Thus, 93 ALOMYbase contigs were identified that encoded predicted peptides with a significant homology to one of the 101 “group 1” or “group 2” *A. thaliana* genes. Expression ratios were calculated for the corresponding ALOMYbase contigs for the sensitive pool and for the resistant pool at each time-point in the time-course experiment as described [34] and compared to those obtained for the *A. thaliana* genes.

The regulation patterns of the 93 ALOMYbase homologs of the *A. thaliana* genes was highly similar in both the sensitive and the resistant *A. myosuroides* pools over the whole time-course (Additional file 3: Table S3). The similarity in the regulation patterns of the 93 ALOMYbase contigs and of their *A. thaliana* counterparts increased with the time elapsed since herbicide application and was highest at 73HAT. At this time-point, 29 ALOMYbase homologs of *A. thaliana* group 1 markers showed a similar regulation in response to the herbicide application in the sensitive and in the resistant pool (Additional file 3: Table S3). Twenty-four of these contigs were up-regulated in both pools. They belonged to the 32 ALOMYbase homologs of the 34 *A. thaliana* “group 1 marker” genes up-regulated after herbicide application. The remaining five contigs were down-regulated in both pools. They belonged to the 12 ALOMYbase homologs of one of the 12 *A. thaliana* “group 1 marker” genes down-regulated after herbicide application. Three additional ALOMYbase homologs of one of the 12 *A. thaliana* “group 1 marker” genes down-regulated after herbicide application also showed a trends in down-regulation in both pools. Among the 49 ALOMYbase homologs of the 55 *A. thaliana* “group 2 marker” genes, 24 had a similar regulation pattern in the sensitive and in the resistant pool at 73HAT. Overall, the regulation pattern of these contigs was most similar to the regulation pattern observed for *A. thaliana* “group 2 marker” genes in response to the ALS inhibitor sulfometuron (Additional file 3: Table S3).

### Transcriptional response to the ALS inhibitors iodosulfuron + mesosulfuron common to both phenotypes (treated *vs*. UT)

Respectively 10,714 and 15,544 contigs were up-regulated or down-regulated in both the resistant and the sensitive pool in at least one treated modality compared to the respective UT modalities (16.3 and 23.7 % of the contigs in ALOMYbase, respectively). The number of contigs regulated in both pools after herbicide application is shown in Fig. 4. Overall, 253 contigs were up-regulated in all treated modalities in both pools, of which 111 could be assigned an InterPro domain annotation (Additional file 3: Table S4). They included four putative cytochrome P450 monooxygenases, two ABC transporters, four glutathione-S-transferases, one peroxidase and 11 UDP-glycosyltransferases. Nine contigs were down-regulated in all treated modalities in both pools, of which one could be assigned an InterPro domain annotation (UDP-glycosyltransferase).

Among the 36 GO Biological Processes significantly enriched (*p*-value < 10^−2^) in up-regulated contigs in both pools (Additional file 3: Table S5), 16 were enriched in several treated modalities. “Multidrug transport” that was mostly enriched in contigs predicted to encode multi antimicrobial extrusion proteins (MatE) and “Branched chain family amino acid biosynthesis” were significantly enriched from 24HAT on. The other enriched GO terms were mostly involved in gene expression regulation, stress response, respiration or amino-acid biosynthesis.

Among the 31 GO Biological Processes significantly enriched (*p*-value < 10^−2^) in down-regulated contigs in both pools (Additional file 3: Table S6), five were enriched in several treated modalities, including “Photosynthesis”. GO terms linked to stress response (e.g., response to oxidative stress, abiotic or biotic stimulus, or wounding) were also enriched in at least one treated modality.

### Specificities of the transcriptional response of the sensitive pool to the ALS inhibitors iodosulfuron + mesosulfuron (treated *vs*. UT)

Respectively 35,423 and 7999 contigs were up-regulated or down-regulated only in the sensitive pool in at least one treated modality compared to UT (54.0 and 12.2 % of the contigs in ALOMYbase, respectively). The number of contigs regulated after herbicide application is shown in Fig. 4. Among the annotated contigs, 23 that had an annotation related to peptide phosphorylation had an expression level higher than in the resistant pool.

Among the 44 GO Biological Processes significantly enriched (*p*-value < 10^−2^) in up-regulated contigs only in the sensitive pool (Additional file 3: Table S7), nine were enriched in several treated modalities. They were involved in gene expression regulation, protein, amino-acid, lipid or carbohydrate metabolism, stress response or cell cycle control. Among these, the GO term most significantly enriched was “Protein amino acid phosphorylation” that included 175 contigs predicted to encode kinases. “Response to oxidative stress” was also highly significantly enriched in three treated modalities, with the 19 up-regulated contigs assigned to this term predicted to encode peroxidases. Other GO terms linked to stress response or amino-acid-metabolism were also enriched (Additional file 3: Table S7).

Among the 13 GO Biological Processes significantly enriched (*p*-value < 10^−2^) in down-regulated contigs (Additional file 3: Table S8), two were enriched in several treated modalities (“Respiratory chain complex IV assembly” and “Oligopeptide transport”). GO terms associated with biosynthesis pathways (including “Asparagine biosynthetic process”), gene expression regulation, photosynthesis or stress response were also significantly enriched.

### Specificities of the transcriptional response of the resistant pool to the ALS inhibitors iodosulfuron + mesosulfuron (treated *vs*. UT)

Respectively 8843 and 27,484 contigs were up-regulated or down-regulated only in the resistant pool in at least one treated modality compared to UT (13.5 and 41.9 % of the contigs in ALOMYbase, respectively). The number of contigs regulated is shown in Fig. 4. Seventy-four contigs were up-regulated in all treated modalities, of which 11 could be assigned an annotation. Even after up-regulation, these contigs had a very low expression level (average RPKM value of 1.25). 193 contigs were down-regulated in all treated modalities, of which 52 could be assigned an annotation. Thirty-seven of these contigs had an expression level lower than in the sensitive pool. The most down-regulated contigs were annotated as one cytochrome P450, one inosine/uridine-preferring nucleoside hydrolase and one ribonuclease.

Among the 24 GO Biological Processes significantly enriched (*p*-value < 10^−2^) in up-regulated contigs in at least one treated modality (Additional file 3: Table S9), three were enriched in several treated modalities (“Photosynthetic electron transport chain”, “Lipid A biosynthetic process” and “ATP synthesis coupled proton transport”). Overall, the enriched GO Biological Processes were involved in gene expression regulation, ATP metabolism, photosynthesis and lipid metabolism (Additional file 3: Table S9).

Among the 28 GO Biological Processes significantly enriched (*p*-value < 10^−2^) in down-regulated contigs in at least one treated modality (Additional file 3: Table S10), four were enriched in several treated modalities (“Lipid metabolic process”, “Protein amino acid phosphorylation”, “Asparagine biosynthetic process” and “Lipid transport”) (Additional file 3: Table S10). GO terms linked to stress response were significantly enriched in one treated modality.

### Contigs showing differences in expression between phenotypes (resistant *vs*. sensitive)

A total of 13,921 contigs were differentially regulated between the resistant pool and the sensitive pool in at least one experimental modality (Additional file 6: Figure S6). Seeking constitutive differences in contig expression between phenotypes identified 339 contigs up-regulated in all modalities in the sensitive pool (Additional file 6: Figure S6), of which 91 could be assigned an annotation. They included six cytochromes P450, one glutathione-S-transferase and one peptidase/thiolesterase. Ten GO Biological Processes mostly involved in stress response and post-translational protein modification were significant enriched in up-regulated contigs in the sensitive pool (Table 2).

**Table 2:** GO Biological Processes significantly enriched in contigs up-regulated in the resistant pool compared to the sensitive pool and in the sensitive pool compared to the resistant pool

Resistant Pool/sensitive			Sensitive Pool/resistant		
Enrichment in contigs up-regulated over the whole time-course					
GO.ID	Term	*p*-value	GO.ID	Term	*p*-value
GO:0006278	RNA-dependent DNA replication	0.00063 (***)	GO:0006950	Response to stress	0.008 (**)
GO:0006952	Defence response	0.01069 (*)	GO:0006468	Protein amino acid phosphorylation	0.015 (*)
GO:0051258	Protein polymerization	0.02974 (*)	GO:0006979	Response to oxidative stress	0.026 (*)
GO:0009664	Plant-type cell wall organization	0.03267 (*)	GO:0032196	Transposition	0.028 (*)
GO:0006950	Response to stress	0.04763 (*)	GO:0043687	Post-translational protein modification	0.028 (*)
			GO:0016310	Phosphorylation	0.029 (*)
			GO:0006793	Phosphorus metabolic process	0.038 (*)
			GO:0006952	Defence response	0.042 (*)
			GO:0006464	Protein modification process	0.043 (*)
Enrichment in contigs up-regulated in all herbicide-treated modalities					
GO:0006952	Defence response	0.00012 (***)	GO:0006313	Transposition, DNA-mediated	0.00039 (***)
GO:0006278	RNA-dependent DNA replication	0.00091 (***)	GO:0006950	Response to stress	0.01159 (*)
GO:0051258	Protein polymerization	0.03208 (*)	GO:0006468	Protein amino acid phosphorylation	0.0252 (*)
GO:0009664	Plant-type cell wall organization	0.03524 (*)	GO:0050896	Response to stimulus	0.02964 (*)
			GO:0006979	Response to oxidative stress	0.03143 (*)
			GO:0043687	Post-translational protein modification	0.04534 (*)
			GO:0016310	Phosphorylation	0.04692 (*)

***, *p*-value < 0.001; **, *p*-value < 0.01; *, *p*-value < 0.05

Conversely, 258 contigs were up-regulated in all modalities in the resistant pool, of which 65 could be assigned an annotation. They included two cytochromes P450. Five GO Biological Processes were significantly enriched in up-regulated contigs in the resistant pool (Table 2). Contigs assigned to these Biological Processes were predicted to encode two RNA-directed DNA polymerases, three reverse transcriptases, three disease resistance proteins, one tubulin, one expansin and one haem peroxidase.

### Identification of candidate NTSR contigs

A commercial herbicide containing iodosulfuron + mesosulfuron was used in our experiments because commercial formulations, and not solely the herbicide molecule(s), exert the pressure selecting for resistance in weed populations. To avoid possible confusing effects due to the formulation, candidate NTSR contigs were selected on the basis of an up-regulation ≥ 2-fold in the resistant pool compared to the sensitive pool at each time-point including UT. Affiliation of candidate contigs to one gene family presumably involved in NTSR was an additional selection criterion. Eleven candidate NTSR contigs were identified. Five were annotated as cytochromes P450, three as glycosyltransferases, one as a peroxidase, one as a helix-loop-helix DNA-binding (transcription factor) and one as a disease resistance protein. The expression level of the 11 contigs was measured in all 42 individual RNA samples used to generate the 14 pooled samples subjected to RNA-Seq (Additional file 3: Table S11). Variation in contig expression that could be substantial was observed between phenotypes, among modalities, and also within modalities (Additional file 7: Figure S7). Despite this variation, all 11 contigs were up-regulated in the resistant pool compared to the sensitive pool at each time-course point, in agreement with ALOMYbase expression data (Additional file 7: Figure S8).

**Fig. 5:**
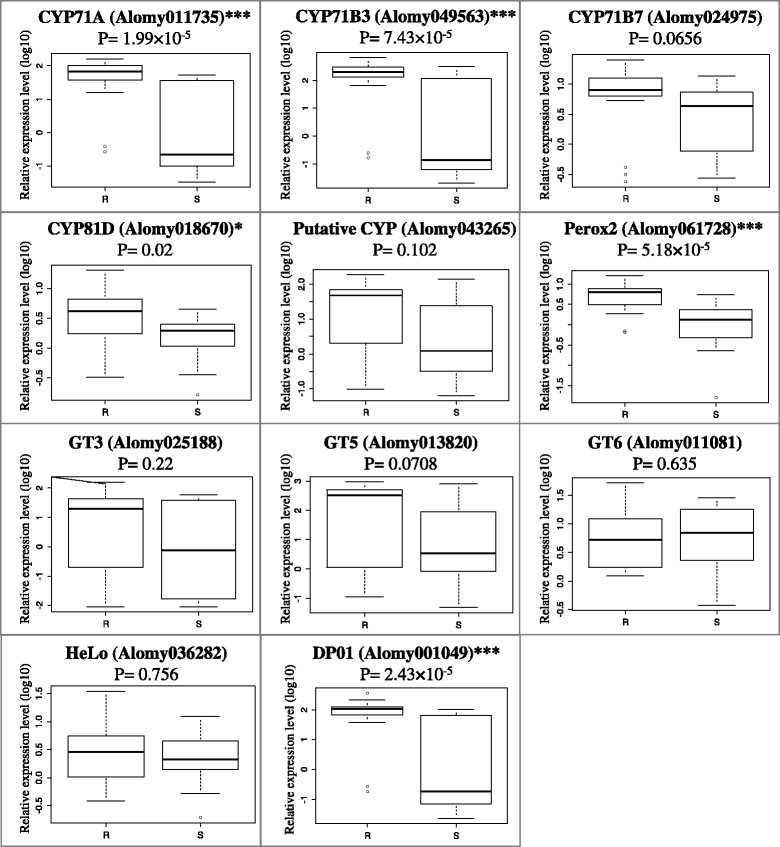
Expression of the 11 candidate NTSR contigs in 29 resistant or sensitive F2 plants. Comparison of the relative expression levels (log10) measured by RT-qPCR of the 11 contigs in F2 plants resistant (R) or sensitive (S) to iodosulfuron + mesosulfuron. The p-values of the Wilcoxon test for pairwise comparison between resistant and sensitive plants are given (*, *p* < 0.05; ***, *p* < 0.001). CYP, cytochrome P450; Perox, peroxidase; GT, glycosyltransferase; HeLo, helix-loop-helix DNA-binding protein; DP, disease resistance protein

The expression levels of the 11 candidate contigs was measured using RT-qPCR in 29 additional, untreated F2 plants: 16 were resistant and 13 sensitive to iodosulfuron + mesosulfuron. Five of the 11 contigs displayed a significantly higher expression level in the resistant plants (Fig. 5). They putatively encoded cytochromes P450 from families 71A, 71B or 81D (referred to hereafter as CYP71A, CYP71B3 and CYP81D), one peroxidase (referred to hereafter as Perox2) and one disease resistance protein (referred to hereafter as DP01). There was a 4919-, 30,989-, 122-, 1008- and 15,764-fold difference in expression between the highest and lowest relative expression levels observed for CYP71A, CYP71B3, CYP81D, Perox2 and DP01, respectively (Additional file 7: Figure S9). This was due to the expression of each of the five contigs being almost undetectable in a few plants, and very high in a few others. For all these plants, both biological replicates showed the same extreme expression level and an expression level of the reference genes similar to those of the other plants analysed. No plant displayed an extreme expression level for more than one contig.

**Fig. 6:**
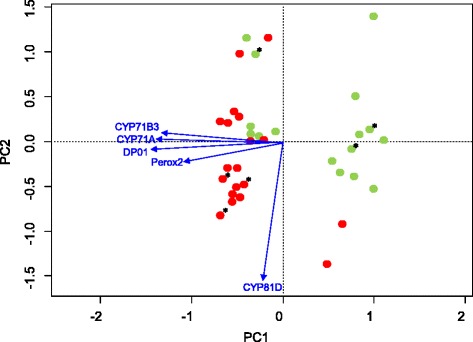
Principal Component Analysis of candidate contig expression data. Expression data was obtained using RT-qPCR for five contigs (CYP71A, CYP71B3, CYP81D, Perox2 and DP01) in 35 *A. myosuroides* F2 plants. Red dots, resistant plants; green dots, sensitive plants. “*” indicate the plants used for RNA-Seq

Although some sensitive plants had expression levels higher than some resistant plants for a given contig, the highest expression levels for each contig were always observed in resistant plants (Additional file 7: Figure S9). The 11, three, nine, 10 and 12 plants most transcribing CYP71A, CYP71B3, CYP81D, Perox2 or DP01, respectively, were all resistant to iodosulfuron + mesosulfuron. Principal Component Analysis implemented using expression data obtained for the five contigs allowed clear separation of most, but not all, resistant F2 plants from the sensitive F2 plants (Fig. 6). Separation occurred along axes determined by the expression levels of two groups of contigs: CYP71A, CYP71B3, Perox2 and DP01 for one axis, and CYP81D for the other (Fig. 6).

Several NTSR genes or candidate genes had previously been identified in grasses (Table 3). Among them, the glutathione-S-transferase AmGSTF1 plays a significant role in *A. myosuroides* NTSR to acetyl-coenzyme A carboxylase (ACCase) inhibitors, a group of leaf-applied herbicides distinct from ALS inhibitors [13, 14]. In *Lolium rigidum*, another major grass weed, four contigs annotated as two cytochromes P450, one monooxygenase and one glycosyl-transferases were also proposed to have a link with NTSR to ACCase inhibitors [15]. More recently, four additional *Lolium sp*. contigs annotated as two cytochromes P450, one glycosyl-transferase and one glutathione-S-transferase were proposed to be associated with NTSR to ALS inhibitors [19]. These contigs were different from those linked to NTSR to ACCase inhibitors in *Lolium rigidum* [19]. Last, one rice cytochrome P450 and one *Echinochloa phyllopogon* cytochrome P450 were also recently demonstrated to confer NTSR to ALS-inhibiting herbicides [16, 17]. A BLASTp search in ALOMYbase identified the most probable ALOMYbase homologs of these genes or contigs (Table 3; E-values between 5.52E-47 and 0.00). The ALOMYbase homologs identified did not include any of the five candidate contigs identified in our work. Overall, RNA-Seq expression patterns of the six ALOMYbase homologs of genes or contigs associated to NTSR to ACCase inhibitors (Table 3) constantly increased with the time after herbicide application in both phenotypes, and their difference in expression between phenotypes was above two-fold in at most one herbicide-treated modality only. This was also true for the homologs of two of the contigs associated to NTSR to ALS inhibitors in *Lolium* sp. The RNA-Seq expression levels of the ALOMYbase homologs of the two other contigs associated to NTSR to ALS inhibitors in *Lolium* sp. was either stable in both phenotypes, or stable in the sensitive phenotype and variable in the resistant phenotype without reaching a two-fold difference in expression between phenotypes (Table 3). RNA-Seq expression level of the ALOMYbase homolog of the two genes associated to NTSR to ALS inhibitors in rice or in *E. phyllopogon* was highest in the sensitive pool. This contig was up-regulated by herbicide application in the sensitive pool only (Table 3). ALOMYbase homologs of known NTSR genes or candidate contigs could therefore not be considered as contigs potentially involved in NTSR to ALS inhibitors in our study.

**Table 3:** ALOMYbase most probable homologs of published NTSR genes or candidate genes identified in grasses and their RNA-Seq expression patterns

Gene^a^	Accession (GenBank)	Reference	Species	Herbicide mode of action^b^	Most probable ALOMYbase homolog			
					Accession	E-value	Expression pattern	
							In the resistant pool	In the sensitive pool
AmGSTF1 (glutathione-S-transferase)	AJ010454	[13, 14]	*A. myosuroides*	ACCase	Alomy081323	1.12E-87	Up over the treated modalities	Up over the treated modalities
Esterase	AJ698940	[12]	*A. myosuroides*	ACCase	Alomy016613	5.18E-140	Up until 36HAT, then down	Up until 36HAT, then down
CYP72A	GAYU01000008	[15]	*Lolium rigidum*	ACCase	Alomy031474	7.90E-179	Up over the treated modalities	Up over the treated modalities
CYP72A	GAYU01000010	[15]	*Lolium rigidum*	ACCase	Alomy009176	2.51E-283	Up over the treated modalities	Up over the treated modalities
Nitronate monooxygenase	GAYU01000016	[15]	*Lolium rigidum*	ACCase	Alomy009031	4.99E-76	Up over the treated modalities	Up over the treated modalities
Glycosyl-transferase	GAYU01000013	[15]	*Lolium rigidum*	ACCase	Alomy080565	5.28E-101	Up over the treated modalities	Up over the treated modalities
CYP72A254	AB755796	[16]	*Echinochloa phyllopogon*	ALS	Alomy010097	1.94E-245	Stable	Up over the treated modalities
CYP72A31	Os01g060220^c^	[17]	*Oryza sativa*	ALS	Alomy010097	3.26E-164	Stable	Up over the treated modalities
GTA (glycosyl-transferase)	LOLSS006751^d^	[19]	*Lolium sp.*	ALS	Alomy047057	1.30E-119	Up over the treated modalities	Up over the treated modalities
GSTA (glutathione-S-transferase)	LOLSS067288^d^	[19]	*Lolium sp.*	ALS	Alomy098098	5.52E-47	Up over the treated modalities	Up over the treated modalities
CYP72A	LOLSS002187^d^	[19]	*Lolium sp.*	ALS	Alomy016824	0.00	Down until 24HAT, then up	Stable
CYP81B1	LOLSS010577^d^	[19]	*Lolium sp.*	ALS	Alomy007987	0.00	Stable but a peak at 24HAT	Stable

^a^CYP, cytochrome P450

^b^Gene or candidate gene conferring NTSR to herbicides inhibiting acetyl-coenzyme A carboxylase (ACCase) or acetolactate synthase (ALS)

^c^Accession in the Rice Genome Annotation Project database, http://rice.plantbiology.msu.edu/

^d^Accession in LOLbase [19]

## Discussion

### ALOMYbase, the first *A. myosuroides* transcriptomic resource

Our main aim was to establish a resource to study transcriptomic patterns in *A. myosuroides* plants resistant or sensitive to leaf-applied herbicides in the absence of herbicide and at the early stages of response to herbicide, using experimental conditions as similar as possible to realistic field conditions. Herbicide damage to plants starts occurring 3 to 8 h after herbicide application (reviewed in [10]). To be efficient, NTSR must be implemented before herbicide damage is irreversible, and must be upheld long enough to allow resistant plants to recover [10]. Accordingly, RNA-Seq data was obtained from a time course experiment ranging from UT until 73HAT. A commercial iodosulfuron + mesosulfuron formulation that is applied in the field was used together with its recommended adjuvant in our experiments, because NTSR is selected for in the field by recurrent applications of not only herbicide molecules, but also associated formulations and adjuvants. Last, resistant and sensitive F2 plants derived from a pairing between a sensitive plant and a resistant plant from a population where NTSR had evolved under herbicide selective pressure were used as starting plant material because using plants with a similar genetic background was expected to facilitate identification of transcriptomic differences related to NTSR [10].

As there is no genomic resource for *A. myosuroides*, the Illumina technology was selected for sequencing because it is the technology of choice for *de novo* transcriptome deep sequencing and assembly without a reference genome [25]. After discarding assembly waste, ALOMYbase contained 65,558 contigs potentially from expressed genes that encoded 32,138 putative peptides (Table 1). This is to be compared to the 95 nucleotide sequences from *A. myosuroides* that had been deposited in GenBank/EMBL to the 5^th^ of June 2015. Our work therefore tremendously increased the sequence data available for *A. myosuroides*.

The contigs in ALOMYbase were assembled for RNA-Seq data obtained from the aerial part of six F2 plants at the vegetative growth stage. Thus, ALOMYbase was not expected to be a comprehensive *A. myosuroides* transcriptomic resource. Accordingly, the completeness of ALOMYbase as estimated by CEGMA analysis was 61.3 %. Considering our starting material and CEGMA estimates of completeness for the transcript sets associated to the five grass genomes (79.8 to 99.2 %), *A. myosuroides* transcriptome representation in ALOMYbase was deemed satisfactory for our purpose.

As *A. myosuroides* genetic variability is high [40], the two parental plants used to generate the F2 population were most likely genetically different. Thus, nucleotide variation was certainly present among the F2 plants used for RNA-Seq, which likely introduced some redundancy among the assembled contigs: more than one contig in ALOMYbase may represent a same unigene and/or several contigs may represent different segments of the same unigene. The precise size of *A. myosuroides* genome is unknown. Estimations based on DNA cell contents give an expected genome size between 1200 and 4330 Mb [41, 42], i.e., a relatively large genome. Large genomes have a low proportion of transcribed sequences encoding proteins (e.g., 5 to 8 % of the large genomes of fully sequenced legume species genomes [38]). From the range of *A. myosuroides* genome size estimates, ALOMYbase sequence data would represent 1.6 to 5.7 % coverage of the *A. myosuroides* genome. Even though redundancy was present in ALOMYbase, this value was deemed acceptable for a transcriptomic resource obtained only from the aerial part of young plants. Five grass species currently have a fully sequenced genome: the weed *B. distachyon* (270 Mb, 25,532 genes encoding proteins), and the crops *O. sativa* (rice, 380 Mb, 40,331 genes), *S. bicolor* (sorghum, 730 Mb, 34,497 genes), *H. vulgare* (barley, 5100 Mb, c.a. 32,000 genes) and *Z. mays* (maize, 2100 Mb, 63,540 genes). The genomes of these species contain 95, 106, 47, 6 and 30 genes encoding proteins per Mb genome, respectively. Depending on *A. myosuroides* genome size estimate, the 32,138 peptides predicted to be encoded by the potentially active contigs in ALOMYbase corresponded to seven to 27 potential genes encoding proteins per Mb genome. Even considering partial transcriptome coverage and occurrence of redundancy, this is in the range of values observed for the grass species with genome sizes similar to that of *A. myosuroides* (barley and maize).

The N50 size value of the contigs in ALOMYbase was 1240 nucleotides (Table 1), a value higher than those obtained for other recent plant *de novo* transcriptome assemblies based on Illumina sequence reads [43, 44] or on a combination of Illumina and 454 pyrosequencing reads [45], and similar to that obtained with 454 sequence reads [15]. The average contig size in ALOMYbase (1046 nucleotides) matched the average length of gene coding sequences in grasses (1000 to 1300 nucleotides [46]).

The predicted peptide content of ALOMYbase was compared to those of the five fully sequenced grass genomes. In total, 30.6 % of the protein families identified using OrthoMCL were shared among ALOMYbase and the five grass genomes (Additional file 2: Figure S3). The five grass genomes shared 41.7 % of the protein families identified. These proportions were in agreement with a previous genome-wide study showing that genome peptide contents was largely shared among grass species, including peptide family representation [47]. While ALOMYbase only represents a part of the total *A. myosuroides* transcriptome, these results suggest a good coverage and a correct representation of the protein families of *A. myosuroides* genome in ALOMYbase. Considering the number of shared protein families, ALOMYbase content was closer to those of *H. vulgare* and *B. distachyon* genomes (Additional file 2: Figure S3). Similarly, rank correlation for the number of predicted peptides assigned to Pfam families was highest between ALOMYbase and *B. distachyon* genome. The six grass species considered belong to three major subfamilies in the *Poaceae*: *Pooideae* (*A. myosuroides*, *H. vulgare* and *B. distachyon*), *Panicoideae* (*Z. mays* and *S. bicolor*) and *Ehrhartoideae* (*O. sativa*) [48]. Thus, similarities in protein family representation are consistent with phylogenetic proximity, as already observed [47].

Peptide annotation identified 2017 ALOMYbase contigs potentially encoding peptides assigned to major families involved in NTSR [5, 10]. Peptides annotated as cytochrome P450 or ABC transporters were particularly abundant in ALOMYbase. This could be due to the probable heterozygous status of the plants sequenced. Heterozygosity has been reported to cause redundancy during assembling, especially in fast-evolving gene families like cytochromes P450 [49]. Other possibilities would be gene evolution via duplication and divergence, a process particularly frequent for cytochromes P450 [50], or occurrence of splice variants, which is expected in plants undergoing a stress because alternative splicing is involved in the regulation of stress response [51]. Even with redundancy present in ALOMYbase, our data suggests *A. myosuroides* genome would be rich in genes encoding proteins potentially involved in NTSR, which could be a reason why NTSR is so widespread and frequent in this species [28].

Particularities in functions such as stress response generally imply particularities in gene expression regulation [47]. A good representation of the transcriptome of the aerial part of *A. myosuroides* young plants and confirmation of the relevance of RNA-Seq-based expression data using RT-qPCR make ALOMYbase a reliable resource to investigate the transcriptomic response to herbicides inhibiting ALS in *A. myosuroides* using RNA-Seq data.

### *A. myosuroides* general response to the ALS-inhibiting herbicides iodosulfuron + mesosulfuron

ALS inhibitors are among the most broadly and frequently used herbicides. Yet, their effects are still not totally elucidated [30]. Most studies addressing plant response to ALS inhibitors were performed on broadleaved plants, particularly *A. thaliana* [33, 34] and *Pisum sativum* [52–54]. Briefly, ALS inhibition is followed by plant growth arrest and the subsequent slow death of treated plants [30]. Application of ALS-inhibiting herbicides causes a rapid induction of specific stress response pathways, including detoxification-related genes [33, 34]. The biosynthesis of branched-chain amino acids (BCAAs: valine, leucine and isoleucine) is interrupted, causing a decrease in the cell contents in free amino-acids, including BCAAs, and a reduction in protein synthesis [30, 55]. This is rapidly followed by an increase in the cellular free amino-acid contents resulting from increased protease-mediated protein degradation and reduced protein synthesis rates [56–58]. Other effects of ALS inhibitors include carbon metabolism impairment leading to an increase in the leaf cell carbohydrate contents and induction of aerobic fermentation [53, 57, 58]. Central energy pathways are also modified in response to oxidative damage [34].

Using a time-course experiment designed as recommended [59] to sample the transcriptome at different times of the day during the early response of *A. myosuroides* to ALS inhibitors allowed analysis of the part of the transcriptomic response to iodosulfuron + mesosulfuron common to resistant and sensitive plants (Additional file 3: Table S5 and S6). The transcriptional response started at 6HAT with an up-regulation of contigs assigned to thiamin biosynthesis process and oxidative stress response. Thiamin had been proposed to be involved in several abiotic and biotic stress responses, including protection against oxidative stress [60–62]. Oxidative stress associated to ALS inhibitor action had been reported to be transient and moderate, and not a cause for plant death [63], which is consistent with the early and transient response observed here in *A. myosuroides* plants. From 24HAT on, there was a strong up-regulation of contigs assigned to protein and BCAAs biosynthesis. This is likely a direct consequence of ALS inhibition. Contigs assigned to “Multidrug transport” were up-regulated from 24HAT on, especially multi antimicrobial extrusion proteins (MATE), which is similar to a previous finding of MATE-encoding contigs being up-regulated in *A. thaliana* in response to three ALS inhibitors [34]. Contigs assigned to “Respiratory gaseous exchange” were also up-regulated. They included seven putative alternative oxidases that are part of the electron transport chain in mitochondria. This may reflect the activation of the alternative respiratory pathway, i.e. aerobic fermentation consecutive to carbohydrate accumulation. This is consistent with previous studies [53, 54, 58, 64, 65]. From 36HAT on, contigs assigned to processes driving gene regulation were up-regulated, while contigs assigned to photosynthesis were down-regulated. This is consistent with plant growth arrest following ALS inhibition. At the latest time-point studied (73HAT), there were few additional changes in the processes enriched in up-regulated contigs. However, there was a drastic increase in the biological processes enriched in down-regulated contigs. In particular, many contigs assigned to various stress response pathways, including oxidative stress response, were down-regulated at 73HAT, as were contigs assigned to oxygen transport and carbon fixation. The effects of ALS inhibitor action on *A. myosuroides* plants reflected by their transcriptional response were consistent with the literature. They can also be considered to reflect the two first phases of herbicide stress response [10, 66]: the initial shock phase when stress-signalling pathways are triggered (from 6HAT to 24HAT), and the acclimation phase when plant resources are re-oriented towards the establishment of defences (from 24HAT to 73HAT).

Similarities in the response to ALS inhibitors were observed between *A. myosuroides* and *A. thaliana*. The expression patterns of the 93 probable ALOMYbase orthologs of *A. thaliana* genes used as markers for the signature of the response to ALS-inhibiting herbicides were established. ALOMYbase orthologs of *A. thaliana* marker genes in groups 1a and 1b showed expression patterns remarkably similar to those of their *A. thaliana* orthologs in both the resistant and the sensitive pools (Additional file 3: Table S3). Similarities in the expression patterns increased with time, with a maximum similarity observed at 73HAT. Our data thus support previous findings that grasses and broadleaved plants share common regulatory mechanisms of gene expression in response to abiotic stresses [67].

From all the foregoing, it is clear that ALOMYbase quantitative and qualitative data are reliable and relevant to study the response to ALS inhibitors in *A. myosuroides*. Plants resistant to iodosulfuron + mesosulfuron because of NTSR reacted to the stress exerted by the herbicide application, with a part of their transcriptomic response being similar to that of sensitive plants and consistent with the effects of ALS inhibitors described in the literature. This is in contrast to a previous study where, following ALS inhibitor application, no transcriptomic changes were observed in plants resistant because of a mutant herbicide-resistant ALS, while their sensitive counterparts showed extensive transcriptomic changes [33]. Here, *A. myosuroides* plants resistant because of NTSR were affected by herbicide action in a first step, but ultimately overcame it, as previously proposed [10]. As expected, determinants of the mechanisms allowing NTSR plants to withstand herbicide action are clearly to be sought in the part of herbicide response that is specific to the resistant phenotype.

### Differential response of resistant and sensitive plants to the ALS-inhibiting herbicides iodosulfuron + mesosulfuron

Overall, there were many more up-regulated contigs than down-regulated contigs in the sensitive plants following herbicide application, while the opposite was observed for the resistant plants (Fig. 4). This could reflect a random response to the herbicide stress in the sensitive plants, while the response in the resistant plants would be more focused and associated with a down-regulation of functions not crucial to overcome ALS inhibitor action. In the sensitive plants, the induction of oxidative stress response seemed more marked, with a specific up-regulation of contigs assigned to this process that could reflect an herbicide stress stronger than in the resistant plants. In the resistant plants, contigs assigned to energy production were up-regulated from 12HAT on, while no significant up-regulation was observed in the sensitive plants. Plant acclimation to stress is an active process that requires extra energy [66]. This observation may thus correspond to an earlier and stronger onset of the phase of acclimation to the herbicide stress in the resistant plants. General analysis of contigs differentially expressed between the resistant and the sensitive plants did not allow identification of biological processes that could directly explain NTSR.

### Candidate NTSR contigs

The primary aim of this work was to identify as many as possible of the contigs presents in plants resistant and/or sensitive to ALS inhibitors before herbicide application and at the early stages of herbicide response, in order to establish a transcriptomic resource as comprehensive as possible to allow subsequent studies of *A. myosuroides* response and NTSR to ALS inhibitors. For this purpose, and because performing RNA-Seq on 42 RNA samples was not affordable, the 42 individual RNA samples studied were sequenced as pooled samples. Transcriptomic data from each pooled sample reflected the average variation in gene expression induced by biological variation among individuals (using three F2 plants per phenotype and per experimental modality) and by the environment (using two clones per F2 plant and per experimental modality). The pooled samples used for RNA-Seq did not contain distinct biological replicates, as is generally recommended to identify contigs differentially expressed among experimental modalities (e.g., [21, 25]). This experimental design was thus not optimal for the purpose of identifying contigs specifically up-regulated in resistant plants that could be candidate NTSR contigs [10], especially because we observed that variation in gene expression could be substantial among the individual samples constituting each pooled sample (Additional file 4: Figure S4; Additional file 7: Figure S7). However, *A. myosuroides* transcriptomic response to ALS inhibitor action obtained from ALOMYbase expression data was fully consistent with the literature. Despite the limitations inherent to our experimental design, this encouraged us to seek potential NTSR determinants using ALOMYbase expression data. Contigs with a constitutive up-regulation in the resistant plants that was maintained during the early phases of the transcriptional response to iodosulfuron + mesosulfuron were targeted because NTSR is expected to be most effective in avoiding irremediable physiological damage if constitutive, and because contigs that could be used for NTSR detection would be most useful if constitutively differentially expressed in resistant plants (i.e., in the absence of herbicide application).

The five contigs identified herein with a potential link to NTSR were predicted to encode three cytochromes P450 (CYP71A, CYP71B and CYP81D), one peroxidase (Perox2) and one disease resistance protein (DP01). Cytochromes P450 in families CYP71 and CYP81 had been shown to be directly involved in herbicide degradation in crop or model species [68]. In particular, wheat CYP71C6 had been shown to catalyse degradation of a range of ALS inhibitors [69]. CYP71A, CYP71B and CYP81D are thus potentially interesting candidate NTSR contigs in *A. myosuroides*. The possible roles of Perox2 and DP01 in NTSR are unclear. ALS inhibitors directly cause oxidative stress, but only as a transient side-effect of ALS inhibition [53]. Thus, a direct role of Perox2 in NTSR is dubious. Peroxidases are involved in a variety of plant physiological processes, including abiotic stress response [70]. Perox2 would rather be a NTSR marker, i.e., a contig which expression level is correlated with NTSR but that has no direct role in NTSR [10]. The same applies to DP01, which potentially encodes a peptide with homologies to a protein involved in response to a biotic stress.

The contigs identified herein differed from NTSR genes or candidate transcripts identified in previous studies. Conversely, previously identified NTSR genes or candidate contigs could not be linked to NTSR in the plants studied in our work (Table 3). In particular, the glutathione-*S*-transferase AmGSTF1 that had been shown to be a major player in *A. myosuroides* NTSR to herbicides inhibiting ACCase [13, 14] was not identified in our work as a potential candidate contig involved in NTSR to ALS inhibitors. The same applies to the ALOMYbase homologs of other genes or candidate transcripts associated to NTSR to ACCase inhibitors [12, 15] (Table 3). However, these genes or contigs were regulated by herbicide application (Table 3). This suggests that they may be involved in the broad response to iodosulfuron + mesosulfuron, but not directly in NTSR to these herbicides. *A. myosuroides* response pathways to ACCase inhibitors and to ALS inhibitors, and thus NTSR to each group of herbicides, may be interrelated, as suggested previously [5]. The ALOMYbase homologs of previously identified genes or contigs associated to NTSR to ALS inhibitors [16, 17, 19] were also not involved in NTSR to ALS inhibitors in the *A. myosuroides* plants studied herein, which is fully consistent with NTSR being underlain by a diversity of mechanisms that vary among species, populations and individuals [5, 6, 9, 10].

In summary, we identified five contigs that are potential NTSR genes or markers. The expression levels of the five contigs varied among the F2 plants analysed: some resistant plants showed a low level of expression for these contigs, and some sensitive plants displayed a high level of expression for some of these contigs. Such variation among individual plants in the expression of genes with a link with NTSR had previously been observed [15, 16, 19, 71]. Principal Component Analysis suggested two groups of NTSR mechanisms could be present in the F2 plants studied, but did not allow separating all resistant plants from all sensitive plants. Our data suggested that the resistant F2 plants studied were not all resistant because of the same NTSR mechanisms, and that not all contigs with a role in NTSR were identified in our study. As all F2 plants studied herein derived from a single parental plant with NTSR to ALS inhibitors, this suggests the occurrence of a set of genes endowing NTSR in the parental plant with NTSR. This is in accordance with previous data showing that NTSR is under polygenic control in *A. myosuroides* [72]. Further investigation is clearly necessary to confirm the link of the five contigs identified with NTSR, and in particular whether the three putative cytochromes P450 have a degrading activity against iodosulfuron and mesosulfuron.

## Conclusions

We obtained RNA-Seq data from the aerial part of young herbicide-resistant and herbicide-sensitive *A. myosuroides* plants that provided substantial transcriptome coverage. This data was assembled to generate ALOMYbase, the first *A. myosuroides* transcriptomic resource. ALOMYbase was used to get insight into the transcriptomic variation occurring in plants resistant or sensitive to iodosulfuron + mesosulfuron following the application of this herbicide. In both resistant and sensitive *A. myosuroides* plants, the transcriptomic response to iodosulfuron + mesosulfuron mirrored known effects of ALS inhibitors and were consistent with the literature data. Striking similarities with the transcriptomic response of *A. thaliana* to ALS inhibitors were observed. Specificities in the response to iodosulfuron + mesosulfuron were observed in the resistant and in the sensitive *A. myosuroides* plants, but our experimental design did not allow identification of processes involved in NTSR. Our data confirmed that gene regulation is at the root of herbicide response and of NTSR. Considering the limitations in our experimental design, contigs potentially involved in constitutive NTSR were tentatively identified. High expression levels of five contigs, of which three potentially encoded cytochromes P450, were correlated with NTSR in the F2 population studied. These contigs are potential NTSR candidates that remain to be fully validated. ALOMYbase is thus a transcriptomic resource for *A. myosuroides* that will be of great use for future research aiming at unravelling the complex, quantitative genetic bases of constitutive and herbicide-induced NTSR to leaf-applied herbicides in this species, understanding its evolution and devising efficient and long-lasting *A. myosuroides* control strategies.

## Methods

### Plant material

*A. myosuroides* is genetically highly variable [40]. Because differences in gene expression can be due to differences in the plant genetic background, genetically homogenised plant material was generated by controlled pairings. One herbicide-resistant plant from the field population CY101 [72] was paired with one herbicide-sensitive plant from the reference population SA98 that exclusively contains herbicide-sensitive plants [72]. F1 progeny seedlings were checked for the absence of ALS mutations endowing herbicide resistance by genotyping as previously described [73]. F1 plants were then transplanted into individual 2 L, plastic pots in a greenhouse (20 °C day, 15 °C night). At the four tiller stage, they were subjected to vegetative propagation: all individual tillers of each plant were separated, transplanted into individual pots and fertilised using a slow-release fertiliser (FEPCOS-BIO, SONOFEP, Saulon-la-Rue, France). This resulted in four clones of each plant at the three-leaf growth stage, at which ALS-inhibiting herbicide application is recommended. All clones had a similar size, height and habit. Herbicide sensitivity of each F1 plant was assessed 72 h after cloning by spraying two clones per plant with the commercial herbicide Atlantis WG (active ingredients: iodosulfuron 0.6 % w/w + mesosulfuron 3 % w/w, Bayer CropScience) at the French field rate (3 g.ha^−1^ iodosulfuron + 15 g.ha^−1^ mesosulfuron) with the adjuvant Actirob B (methylated rapeseed oil, Bayer CropScience, 1 L.ha^−1^). In the following, the herbicide mixture applied will be referred to as “iodosulfuron + mesosulfuron”. Herbicide application was as described [72]. The two remaining clones per F1 plant were sprayed with water (untreated control). Four clones of plants from the reference population SA98 were included in the spraying experiment to check herbicide application efficacy. Visual phenotype rating was performed 4 weeks after treatment, when the reference sensitive control plants were clearly dead [72]. Plants were assigned to three phenotypes classes: highly resistant (sprayed clones identical to the untreated clones), moderately resistant (treated clones survived herbicide application but displayed a reduced growth and/or herbicide symptoms compared to the untreated clones) and sensitive (treated clones killed), as previously described [72]. One water-treated clone of one sensitive F1 plant and one water-treated clone of one resistant F1 plant were then paired, yielding a F2 population. This F2 population was used in all the subsequent experiments.

Visual phenotype rating of the F2 plants using iodosulfuron + mesosulfuron was as before. All clones had a similar size, height and habit. F2 plants with contrasted phenotypes (i.e., three highly resistant and three sensitive plants) were selected for transcriptome analysis using RNA-Seq.

### Sample collection

Our aim was to identify as many transcripts present in each phenotype before and during the early phase of response to ALS inhibitors as possible, considering that (i) herbicide damage to plants starts occurring 3 to 8 h after herbicide application and (ii), to be efficient, NTSR must be implemented before herbicide damage is irreversible, and must be upheld long enough to allow resistant plants to recover (reviewed in [10]). Many stress responsive genes are under rhythmic regulation and show time-of-day dependence in their regulation: transcriptome sampling during an off-peak regulation period may thus lead to an incomplete representation of the herbicide response [59]. Accordingly, to capture as much as possible of the transcriptomic herbicide response to ALS inhibitors as possible, a time-course experiment was performed containing a range of time-points positioned at different times of the day [59].

Untreated clones from each of the six F2 plants intended for RNA-Seq were grown until they had developed 16 tillers, and subsequently split into individual tillers. Each clone was transplanted into an individual pot as before. All clones had a similar size, height and habit. Two clones (biological replicates) intended for RNA-Seq were used per plant in each of six time-points (i.e. 12 clones per plant in total): untreated (UT), 6, 12, 24, 36 and 48 h after treatment (HAT). The four remaining clones of each plant were not sampled for RNA-Seq: two clones sprayed with iodosulfuron + mesosulfuron (phenotype control) and two clones sprayed with water (untreated control) together with the clones intended for the time-course experiment were used to check plant phenotype (i.e., resistant or sensitive) 4 weeks after treatment. Herbicide application was as before, and included clones from reference sensitive plants as a check for herbicide application efficacy. An additional experimental modality consisting of samples collected 73HAT was included in the RNA-Seq experiment. This modality came from a second time-course experiment performed 8 weeks after the first one following the same procedure. It contained two clones from each of the same six plants used in the first time-course that were treated with iodosulfuron + mesosulfuron as before. The 73HAT modality was included to identify contigs expressed at a later time-point after herbicide application and to check the trends in gene expression patterns observed in the first time-course.

One sample collected for RNA extraction consisted of the aerial part of the two clones of one given F2 plant at one given time-point. Plant material was immediately placed into liquid nitrogen in order to avoid RNA degradation and/or induction of plant response to wounding, and stored at −80 °C until plant phenotype had been checked 4 weeks after herbicide application. A total of 42 samples were thus collected (six plants with two clones each × seven time-points, including 73HAT).

### RNA extraction and sequencing

Total RNA from the 42 samples was extracted using the RNeasy Plant Mini Kit (Qiagen, Courtaboeuf, France) according to the manufacturer’s instructions. Potential genomic DNA contaminations were removed using the RNase-Free DNase Set (Qiagen).

Our aim was to generate a transcriptomic resource including as many as possible of the transcripts present in the aerial part of plants with a resistant or a sensitive phenotype before herbicide application and at the early stages of herbicide response. Pooled RNA samples were sequenced because we considered there could be differences in expression patterns among plants. Pooling samples was expected to allow capture of a maximum of transcripts by sequencing a minimum of samples. One pooled sample consisted of an equimolar mixture of the individual RNA samples extracted from the six biological replicates per phenotype in a given experimental modality in the time-course experiment (i.e., two clones for each of the three resistant or sensitive F2 plants in each experimental modality). A total of 14 pooled RNA samples were thus produced and used for RNA-sequencing.

RNA quality control was carried out on an Agilent 2100 Bioanalyzer (Waldbroon, Germany) according to the manufacturer’s instructions. The RNA integrity number was between 8 and 9 for all pooled samples. A non-normalised cDNA library was generated for each of the 14 pooled samples. Library preparation and sequencing were performed by Fasteris (Plan-les-Ouates, Switzerland) using Illumina protocols: 1 μg RNA from each of the 14 pooled samples was processed using TruSeq RNA Sample Prep kit (Illumina). Transcripts were purified and fragmented by zinc breaking using mRNA-SEQ kit (Illumina). Double-stranded cDNA fragments were prepared with random primers and RNaseH. Libraries were subjected to 15 PCR cycles. Fragments were purified on agarose gel to recover fragments with inserts in the range of 160–240 base pairs. All libraries were sequenced in two independent flow cell lanes (technical replicates) on an Illumina HiSeq2000 system. Sequencing was carried out following the manufacturer’s instructions for the generation of single-end, 100-base long reads. Sequence data was extracted using the CASAVA 1.8.1 pipeline (Illumina). The sequence reads from all 14 libraries were analysed according to Fasteris quality control, using an indexed PhiX reference sequence on each call lane to estimate sequencing error.

### *De novo* transcriptome assembly and annotation

The F2 plants used for RNA-Seq were issued from genetically unrelated parental plants. Nucleotide variation among these plants was thus expected. This was anticipated to complicate *de novo* transcriptome assembly. A custom iterative procedure was thus designed to handle heterozygosity and sequencing errors. The first step of the assembling procedure was based on iterative Velvet runs [74] configured with stringent parameters (k-mer ranging from 41 to 85 with a step of 4, and a max_divergence set to 0.01). Reads were considered as stranded (−strand_specific parameter) even if the actual data were not. At each iteration, the contig sequences assembled during the previous iteration were integrated as long reads (−long). This highly time-consuming procedure generated a set of accurate long reads. As these data were very similar in quantity and quality to Sanger EST data, an iterative pipeline was developed that integrated a “containment clustering”-like program (nrcl-like program) and the cap3 assembler [75] that is widely used to assemble Sanger ESTs. Each iteration in the pipeline combined a nrcl run that removed redundancy by including short redundant contigs into longer contigs to a cap3 assembly that merged contigs. At each iteration, less and less stringent parameters were used because each iteration added constraints to the subsequent one. The thresholds used both for nrcl and cap3 were an “Identity percentage” ranging from 92 to 99 %, a length of the overhang of 20 nucleotides, an “High-scoring Segment Pair” (HSP) length of 100 and 75 nucleotides, respectively.

The quality of the assembly was manually checked on a set of contigs expected to correspond to single-copy genes. The assembly parameters were selected so that single consensus sequences were obtained for this set of genes. Putative coding sequences were sought in the assembled contigs using FrameDP trained on *A. thaliana*, *B. distachyon* and *M. truncatula* proteomes [76]. Putative peptide annotation was performed using an automatic InterproScan analysis [77] and included Gene Ontology (GO) terms, Enzyme Commission (EC) codes and Pfam domains. Similarity searches for peptide annotation were also conducted against TAIR10, *Brachypodium distachyon* proteome, Swiss-Prot and TrEMBL using the BLASTp algorithm with an E-value < 10^−5^. Additional Pfam domain assignation was performed using the Pfam-A 27.0 database and the hmmsearch program [78].

### Comparison with other grass species (*Poaceae*)

The predicted peptide contents of *A. myosuroides* transcriptomic database (ALOMYbase) was compared to those of the five grass (*Poaceae*) species for which a full genome sequence is available: the weed *Brachypodium distachyon* and the crop species *Hordeum vulgare* (barley), *Zea mays* (maize), *Oryza sativa* (rice) and *Sorghum bicolor* (sorghum). All data were collected from Phytozome v.9.0, except *H. vulgare* data that were collected from the MIPS database (http://mips.helmholtz-muenchen.de/plant/barley). InterProScan annotation was performed anew for the five grass genomes using the same procedure as for ALOMYbase to homogenise functional annotation and more accurately identify peptides assigned the same annotations in all species. Comparison of the Pfam families present in ALOMYbase and in the five grass genomes was performed using OrthoMCL [79] with an 80 % match cutoff threshold for ortholog clustering. A Pfam family was considered present in one species if at least one peptide in this species was assigned to this family. The distribution of the predicted peptides among Pfam families was compared among ALOMYbase and the grass genomes.

The completeness of *A. myosuroides* transcriptome assembly was assessed using CEGMA VM (Core Eukaryotic Genes Mapping Approach) that checks the presence of a core protein set consisting of 248 highly conserved proteins found in a wide range of eukaryotes [39] (http://korflab.ucdavis.edu/datasets/cegma). For comparison purpose, CEGMA analysis was also performed on the set of transcripts available for each of the five grass species with a full genome.

### Contig expression analysis

To measure gene expression in each experimental modality, all the corresponding sequence reads were mapped against the assembled contigs using the glint software (Faraut & Courcelle; http://lipm-bioinfo.toulouse.inra.fr/download/glint/, unpublished) configured to keep only the best scoring reads with the following parameters: no gap in alignment, maximum mismatch number = 5, minimum score = 24 and length of each read aligned ≥ 50 %. Redundancy was expected in the assembly. Thus, a given read in an experimental modality could be mapped to different contigs, so that differential expression analysis was not biased by redundancy. The total number of reads mapped per contig was computed for each experimental modality and normalized using the RPKM method [80].

### RT-qPCR validation of RNA-Seq expression patterns using the original RNA samples

Primers were designed based on the sequence of 21 contigs showing *in silico* expression levels stable or variable among experimental modalities, and checked with NetPrimer (Premier Biosoft, Palo Alto, California). Amplification specificity and qPCR efficacy were checked for each contig as described [81]. Primer pairs retained for contig expression measurement had an efficiency value between 80 and 110 % (Additional file 3: Table S2). The expression level of the 21 contigs was measured in each of the 42 individual RNA samples used to create the 14 pooled samples subjected to RNA-Seq. Analyses were performed in duplicate (technical replicates) for each sample. Reverse transcription was performed using the Masterscript RT-PCR System kit (5 PRIME, Hamburg, Germany) starting from 5 μg total RNA. The StepOnePlus™ Real-Time PCR System Thermal Cycling Block (Applied Biosystems, Foster City, USA) was used to perform qPCRs in fast optical 0.1 mL, 96-well reaction plates (MicroAmp™, Applied Biosystems, Cheshire, UK). Reaction mixes, PCR programs, contig expression measurement and normalization with three previously validated reference genes using a five-point standard dilution curve were as described [81].

### Identification of differentially expressed contigs and GO term enrichment

Contigs with differences in expression among experimental modalities were identified via pairwise differential expression analysis using the DESeq package in the R statistical software [82]. The following criteria were used to identify differentially expressed contigs: change in expression ≥ 2-fold and RPKM ≥ 1.8 in at least one of the experimental modalities.

The TopGO package [83] was subsequently used to identify the Gene Ontology (GO [84]) biological processes significantly enriched in contigs with a differential regulation among experimental modalities, considering contigs with a minimum length of 400 nucleotides, an RPKM value ≥ 1.8 in at least one experimental modality and a difference in expression ≥ 2-fold between the experimental modalities compared. The elim method was implemented to eliminate local similarities and dependencies between GO terms [83]. The transcriptomic response to iodosulfuron + mesosulfuron of each phenotype (resistant or sensitive) over time was assessed by performing TopGO pairwise comparisons between each treated experimental modality and the corresponding UT modality. To identify phenotype-associated differences in herbicide response, pairwise comparisons were performed between phenotypes at each time point.

### Candidate NTSR contig identification

Contigs with a possible role in NTSR were identified after comparison of the transcriptomic patterns of the resistant and the sensitive pool over the time-course experiment. The expression level of candidate contigs was measured in the original 42 individual RNA samples using RT-qPCR as before. For further validation, the expression level of the candidate contigs was also measured in untreated clones of 29 additional F2 plants that had been characterised using iodosulfuron + mesosulfuron. Briefly, each plant was split into six clones. Two clones intended for RNA extraction were collected as before immediately prior to treatment. Iodosulfuron + mesosulfuron was applied on two clones per plant as before (phenotype control). The last two clones were water-treated controls. 4 weeks after herbicide application, F2 plants which treated clones were killed or survived and healthily grew were rated sensitive or resistant, respectively.

### Data access

The raw reads have been deposited in the NCBI Sequence Read Archive (SRA) database (BioProject PRJNA234492, SRR1139294 to SRR1139349). The 65,558 contigs and the corresponding 32,138 predicted peptides sequences in ALOMYbase are available at https://iant.toulouse.inra.fr/A.myosuroides2013?download=1.

## Additional files

Additional file 1: Figure S1. Number of Illumina sequence reads obtained for each experimental modality. **Figure S2.** Percentages of contigs with at least one mapped read and % of reads mapped to a contig for each experimental modality. Mapping data for libraries derived from the resistant pool (R, red and brown bars) and from the sensitive pool (S, blue bars). UT, untreated; xHAT, x hours after herbicide application. (DOCX 80 kb) Additional file 2: Figure S3. OrthoMCL analysis results showing groups of peptides shared among ALOMYbase and grass genomes. The six-way Venn diagram shows the number of OrthoMCL groups of peptides shared among ALOMYbase and *B. distachyon*, *H. vulgare*, *Z. mays*, *O. sativa* and/or *S. bicolor.* An 80 % match cutoff threshold was implemented for ortholog clustering. (PPTX 104 kb) Additional file 3: Table S1. Pfam families most represented in ALOMYbase. **Table S2.** Primers used for RT-qPCR validation of ALOMYbase RNA-Seq expression data. **Table S3.** Expression patterns of the ALOMYbase contigs that are the most likely homologs of transcripts identified in *Arabidopsis thaliana* as herbicide response markers allowing to differentiate the action of five herbicides, including four ALS inhihitors (IMAZ, imazapyr; PRIM, primisulfuron; CLOR, clorasulam; SULF, sulfometuron). **Table S4.** Functional annotation of the 253 contigs up-regulated in all herbicide-treated modalities in both the resistant and the sensitive pools. **Table S5.** GO Biological Processes significantly enriched in contigs up-regulated in both phenotypes (resistant and sensitive pools) after herbicide application. **Table S6.** GO Biological Processes significantly enriched in contigs down-regulated in both phenotypes (resistant and sensitive pools) after herbicide application. **Table S7.** GO Biological Processes significantly enriched in contigs up-regulated after herbicide application only in the sensitive pool. **Table S8.** GO Biological Processes significantly enriched in contigs down-regulated after herbicide application only in the sensitive pool. **Table S9.** GO Biological Processes significantly enriched in contigs up-regulated after herbicide application only in the resistant pool. **Table S10.** GO Biological Processes significantly enriched in contigs down-regulated after herbicide application only in the resistant pool. **Table S11.** Primers used for RT-qPCR measurement of candidate NTSR contig expression. (XLSX 125 kb) Additional file 4: Figure S4. RT-qPCR expression patterns of the 21 contigs used for RNA-Seq expression data validation. The expression values were measured in each of the three resistant F2 plants (R1, R2, R3; red bars) and each of the three sensitive F2 plants (S1, S2, S3; green bars) used for RNA-Seq in each experimental modality. RT-qPCR expression data is normalised using three reference genes. (PPTX 876 kb) Additional file 5: Figure S5. RT-qPCR validation of the RNA-Seq expression patterns of 21 contigs. The expression values were computed for the resistant (R, black bars) or the sensitive pool (S, white bars) for each experimental modality. Normalised expression values were measured by RT-qPCR and averaged for the F2 three plants in each pool (A) or were computed as RPKM values from RNA-Seq data (B). Pearson’s coefficient correlation computed between RT-qPCR and RNA-Seq expression patterns are given in red. (PPTX 148 kb) Additional file 6: Figure S6. Number of contigs differentially regulated between phenotypes. Six-way Venn diagrams show the number of contigs up-regulated at each time-point in the sensitive pool compared to the resistant pool (A) or in the resistant pool compared to the sensitive pool (B). UT, untreated; xHAT, x hours after herbicide treatment. (PPTX 111 kb) Additional file 7: Figure S7. RT-qPCR expression patterns of 11 candidate NTSR contigs. The expression values were measured in each of the three resistant F2 plants (R1, R2, R3; red bars) and each of the three sensitive F2 plants (S1, S2, S3; green bars) in each experimental modality used for RNA-Seq. RT-qPCR expression data is normalised using three reference genes. CYP, cytochrome P450; Perox, peroxidase; GT, glycosyltransferase; HeLo, helix-loop-helix DNA-binding protein; DP, disease resistance protein. **Figure S8.** RT-qPCR validation of the RNA-Seq expression patterns of the 11 candidate NTSR contigs. The expression values were computed for the resistant (R, black bars) or the sensitive pool (S, white bars) for each experimental modality. Normalised expression values were measured by RT-qPCR and averaged for the three F2 plants in each pool (A) or were computed as RPKM values from RNA-Seq data (B). Pearson’s coefficient correlation computed between RT-qPCR and RNA-Seq expression patterns are given in red. CYP, cytochrome P450; Perox, peroxidase; GT, glycosyltransferase; HeLo, helix-loop-helix DNA-binding protein; DP, disease resistance protein. **Figure S9.** Individual relative expression levels (log10) measured by RT-qPCR of the five candidate NTSR contigs showing a higher expression in resistant plants plotted by increasing value for 35 *A. myosuroides* F2 plants. Green, sensitive plants; red, resistant plants. (*) indicate the three resistant and the three sensitive F2 plants used for RNA-Seq. (PPTX 1295 kb)

## Abbreviations

ALS: Acetolactate-synthase
ACCase: Acetyl-coenzyme A carboxylase
NTSR: Non-target-site resistance
RNA-Seq: RNA-sequencing
RPKM: Reads per kilobase per million reads
RT-qPCR: Reverse transcription followed by quantitative PCR
