# Supplementary material for: ALOMYbase, a resource to investigate non-target-site-based resistance to herbicides inhibiting acetolactate-synthase (ALS) in the major grass weed Alopecurus myosuroides (black-grass)
Source: BMC Genomics. 2015 Aug 12;16(1):590. doi: 10.1186/s12864-015-1804-x (PMC4534104; doi:10.1186/s12864-015-1804-x)
Supplement: Additional file 1: Figure S1. — Number of Illumina sequence reads obtained for each experimental modality. Figure S2. Percentages of contigs with at least one mapped read and % of reads mapped to a contig for each experimental modality. Mapping data for libraries derived from the resistant pool (R, red and brown bars) and from the sensitive pool (S, blue bars). UT, untreated; xHAT, x hours after herbicide application. (DOCX 80 kb) [file 12864_2015_1804_MOESM1_ESM.docx]

Figure S1: Number of Illumina sequence reads obtained for each experimental modality.


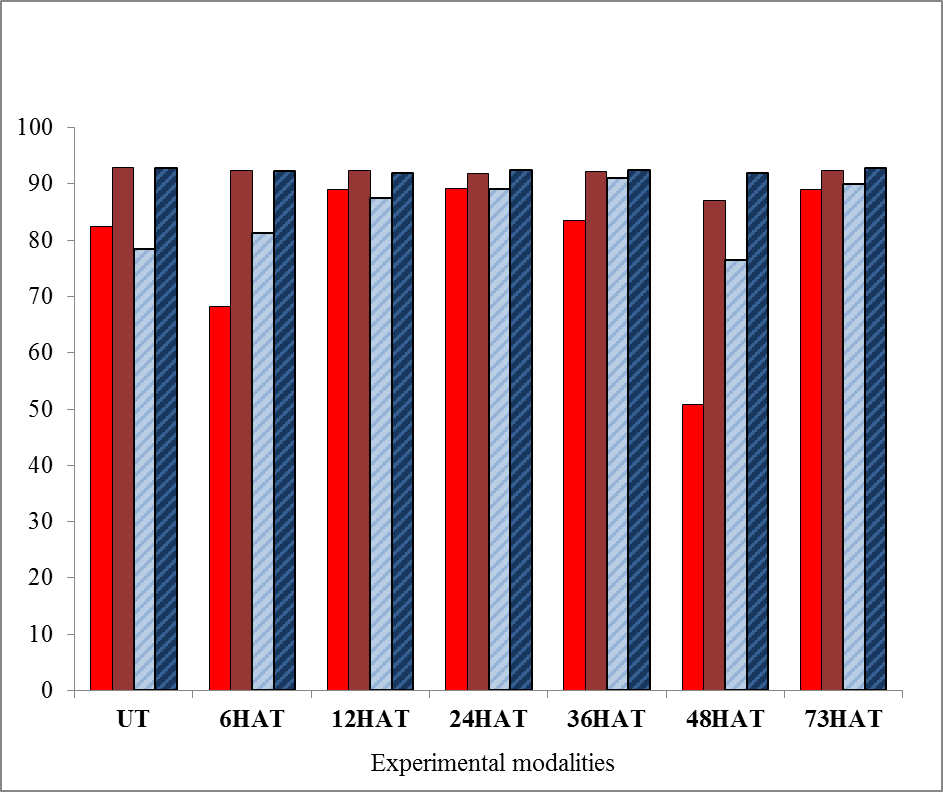

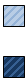


% contigs mapped

% reads mapped


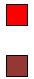


% contigs mapped

% reads mapped

Sensitive pool

Resistant pool

%

Figure S2: Percentages of contigs with at least one mapped read and % of reads mapped to a contig for each experimental modality. Mapping data for libraries derived from the resistant pool (R, red and brown bars) and from the sensitive pool (S, blue bars). UT=untreated, xHAT= x hours after herbicide application.
