# Supplementary material for: ALOMYbase, a resource to investigate non-target-site-based resistance to herbicides inhibiting acetolactate-synthase (ALS) in the major grass weed Alopecurus myosuroides (black-grass)
Source: BMC Genomics. 2015 Aug 12;16(1):590. doi: 10.1186/s12864-015-1804-x (PMC4534104; doi:10.1186/s12864-015-1804-x)
Supplement: Additional file 2: Figure S3. — OrthoMCL analysis results showing groups of peptides shared among ALOMYbase and grass genomes. The six-way Venn diagram shows the number of OrthoMCL groups of peptides shared among ALOMYbase and B. distachyon, H. vulgare, Z. mays, O. sativa and/or S. bicolor. An 80 % match cutoff threshold was implemented for ortholog clustering. (PPTX 104 kb) [file 12864_2015_1804_MOESM2_ESM.pptx]

## Slide 1
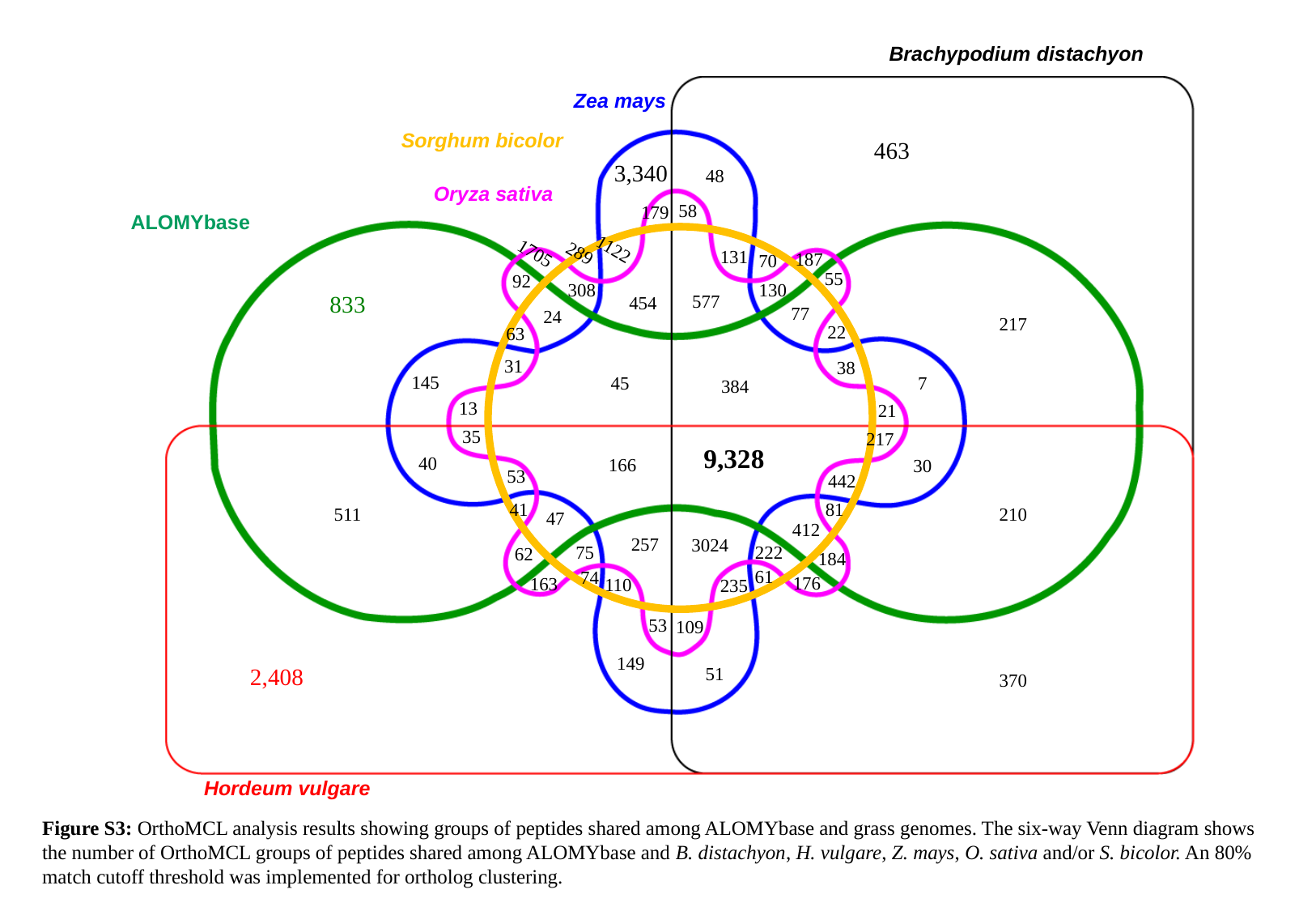

Brachypodium distachyon
Zea mays
Sorghum bicolor
463
3,340
48
Oryza sativa
58
179
ALOMYbase
1122
289
1705
131
187
70
55
92
130
308
833
577
454
77
24
217
22
63
31
38
145
45
7
384
13
21
35
217
9,328
40
166
30
53
442
41
81
511
210
47
412
257
3024
75
222
62
184
61
74
176
163
110
235
53
109
149
2,408
51
370
Hordeum vulgare
Figure S3: OrthoMCL analysis results showing groups of peptides shared among ALOMYbase and grass genomes. The six-way Venn diagram shows the number of OrthoMCL groups of peptides shared among ALOMYbase and B. distachyon, H. vulgare, Z. mays, O. sativa and/or S. bicolor. An 80% match cutoff threshold was implemented for ortholog clustering.
