# Supplementary material for: ALOMYbase, a resource to investigate non-target-site-based resistance to herbicides inhibiting acetolactate-synthase (ALS) in the major grass weed Alopecurus myosuroides (black-grass)
Source: BMC Genomics. 2015 Aug 12;16(1):590. doi: 10.1186/s12864-015-1804-x (PMC4534104; doi:10.1186/s12864-015-1804-x)
Supplement: Additional file 4: Figure S4. — RT-qPCR expression patterns of the 21 contigs used for RNA-Seq expression data validation. The expression values were measured in each of the three resistant F2 plants (R1, R2, R3; red bars) and each of the three sensitive F2 plants (S1, S2, S3; green bars) used for RNA-Seq in each experimental modality. RT-qPCR expression data is normalised using three reference genes. (PPTX 876 kb) [file 12864_2015_1804_MOESM4_ESM.pptx]

## Slide 1
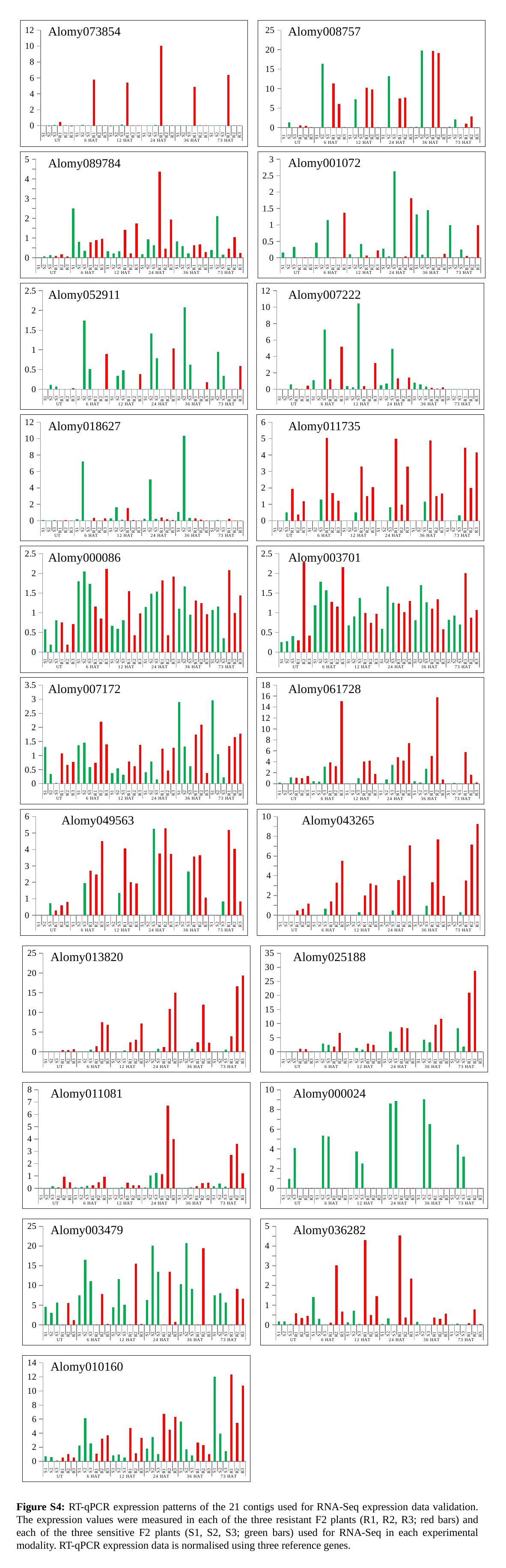

Alomy008757
### Chart
| Category | |
|---|---|
| S1 | 0.00477492872737172 |
| S2 | 0.000984994361400252 |
| S3 | 0.10554507039748724 |
| R1 | 0.44694478970431956 |
| R2 | 0.016412639308409045 |
| R3 | 0.001883826791590026 |
| S1 | 0.02141984597760475 |
| S2 | 0.08455687220102427 |
| S3 | 0.022119238513334916 |
| R1 | 5.792053062510459 |
| R2 | 0.021393815107918376 |
| R3 | 0.05079684839952614 |
| S1 | 0.0015599194666280252 |
| S2 | 0.013978329279308713 |
| S3 | 0.16159830352437327 |
| R1 | 5.426717910369548 |
| R2 | 0.020254797648436438 |
| R3 | 0.047156877802092566 |
| S1 | 0.0 |
| S2 | 0.0399179327903935 |
| S3 | 0.06704259766489475 |
| R1 | 10.011069305103645 |
| R2 | 0.034053255187318034 |
| R3 | 0.045847049163743915 |
| S1 | 0.014658390597066648 |
| S2 | 0.008878991123834315 |
| S3 | 0.020504764207782675 |
| R1 | 4.876905460535773 |
| R2 | 0.040851610401370524 |
| R3 | 0.01617652870094516 |
| S1 | 0.017848963289199105 |
| S2 | 0.01662234677872912 |
| S3 | 0.0052058937423482155 |
| R1 | 6.359550742265452 |
| R2 | 0.011838270611625906 |
| R3 | 0.018482255830998787 |Alomy073854
### Chart
| Category | |
|---|---|
| S1 | 0.02722680598332944 |
| S2 | 1.35589444274241 |
| S3 | 0.0 |
| R1 | 0.6093562558932827 |
| R2 | 0.40239822081477405 |
| R3 | 0.11481964584059635 |
| S1 | 0.004057093057316535 |
| S2 | 16.420666401249473 |
| S3 | 0.12639371924953877 |
| R1 | 11.353952259967393 |
| R2 | 6.008663850847969 |
| R3 | 0.07729328064979085 |
| S1 | 0.012249928496694042 |
| S2 | 7.290092595118803 |
| S3 | 0.004855041449139737 |
| R1 | 10.228755946966322 |
| R2 | 9.736076420757811 |
| R3 | 0.0107952293987848 |
| S1 | 0.11772599452656457 |
| S2 | 13.165603699522379 |
| S3 | 0.04992481071778098 |
| R1 | 7.476761832544372 |
| R2 | 7.735792038803257 |
| R3 | 0.10262324571220571 |
| S1 | 0.20185801821826987 |
| S2 | 19.776065234574393 |
| S3 | 0.05767104357344319 |
| R1 | 19.710935209089232 |
| R2 | 19.06862979650516 |
| R3 | 0.053470928760837255 |
| S1 | 0.2224243562359067 |
| S2 | 2.1442990307577774 |
| S3 | 0.0 |
| R1 | 1.0202254880911337 |
| R2 | 2.8379723669296237 |
| R3 | 0.00047467610293025904 |
### Chart
| Category | |
|---|---|
| S1 | 0.16465016529230916 |
| S2 | 0.0 |
| S3 | 0.3239755232474089 |
| R1 | 0.0 |
| R2 | 0.0027745848610502485 |
| R3 | 0.014813094875221288 |
| S1 | 0.4594582782069594 |
| S2 | 0.0 |
| S3 | 1.1436280276877004 |
| R1 | 0.0008350109172809377 |
| R2 | 0.0 |
| R3 | 1.3661809497520627 |
| S1 | 0.10926626939541129 |
| S2 | 0.0 |
| S3 | 0.42360987091778357 |
| R1 | 0.07217962479606292 |
| R2 | 0.0 |
| R3 | 0.22474263542657455 |
| S1 | 0.2794241869808576 |
| S2 | 0.03583417576066348 |
| S3 | 2.6304940526955076 |
| R1 | 0.0019631896595532967 |
| R2 | 0.04144207397459613 |
| R3 | 1.810999834787428 |
| S1 | 1.3188072623451785 |
| S2 | 0.09494533877426421 |
| S3 | 1.4478087340897858 |
| R1 | 0.003737834725145451 |
| R2 | 0.01193960536796524 |
| R3 | 0.11625905572843226 |
| S1 | 0.9950379172188755 |
| S2 | 0.0008331019431124269 |
| S3 | 0.2507368746978024 |
| R1 | 0.049111284830365864 |
| R2 | 0.01133594975220299 |
| R3 | 0.9868863629269048 |Alomy001072
### Chart
| Category | |
|---|---|
| S1 | 0.036356066583957215 |
| S2 | 0.06343049265516973 |
| S3 | 0.12511560093385524 |
| R1 | 0.08757880342649722 |
| R2 | 0.18767731752320793 |
| R3 | 0.0789633715634178 |
| S1 | 2.5093720802300203 |
| S2 | 0.8044945680666902 |
| S3 | 0.34672791447531887 |
| R1 | 0.7916970414419947 |
| R2 | 0.8851071166642989 |
| R3 | 0.9524234104201064 |
| S1 | 0.32279656540671864 |
| S2 | 0.22690690631431898 |
| S3 | 0.32662959677335657 |
| R1 | 1.4072140234850625 |
| R2 | 0.221453849585486 |
| R3 | 1.750033940588203 |
| S1 | 0.1709781603723861 |
| S2 | 0.9467259645147785 |
| S3 | 0.6369428788445518 |
| R1 | 4.3750042168135606 |
| R2 | 0.4630719535059687 |
| R3 | 1.9332493452864117 |
| S1 | 0.8368461196253856 |
| S2 | 0.58824013141698 |
| S3 | 0.2258679514644256 |
| R1 | 0.6346847281373066 |
| R2 | 0.6711305223074252 |
| R3 | 0.2892246137846446 |
| S1 | 0.3975228078288173 |
| S2 | 2.1186949328102025 |
| S3 | 0.1528869186240445 |
| R1 | 0.4539102864007245 |
| R2 | 1.0391419874467518 |
| R3 | 0.24754314656958024 |Alomy089784
### Chart
| Category | |
|---|---|
| S1 | 0.0 |
| S2 | 0.10820235310923099 |
| S3 | 0.06643591764892726 |
| R1 | 0.0 |
| R2 | 0.0 |
| R3 | 0.02198045871745428 |
| S1 | 0.0 |
| S2 | 1.7474764918571803 |
| S3 | 0.5149743737411566 |
| R1 | 0.0 |
| R2 | 0.0 |
| R3 | 0.8912017584188939 |
| S1 | 0.0 |
| S2 | 0.3380639865016048 |
| S3 | 0.48256895963605784 |
| R1 | 0.0 |
| R2 | 0.0 |
| R3 | 0.3791479165767584 |
| S1 | 7.828863838228187e-05 |
| S2 | 1.4178999699689214 |
| S3 | 0.7894987241458119 |
| R1 | 0.0 |
| R2 | 0.0 |
| R3 | 1.0345225906232047 |
| S1 | 0.0 |
| S2 | 2.083331053474382 |
| S3 | 0.627404464669395 |
| R1 | 0.0 |
| R2 | 0.0 |
| R3 | 0.17354266724448672 |
| S1 | 0.0 |
| S2 | 0.9465010358487634 |
| S3 | 0.33593741424204054 |
| R1 | 0.0 |
| R2 | 0.0006196176370352417 |
| R3 | 0.5904847533077731 |Alomy052911
### Chart
| Category | |
|---|---|
| S1 | 0.00900838398262631 |
| S2 | 0.04487582701465071 |
| S3 | 0.5817106527724896 |
| R1 | 0.05387098026717818 |
| R2 | 0.00516414945440379 |
| R3 | 0.43621618638932746 |
| S1 | 1.1130895850654456 |
| S2 | 0.0012541742682438987 |
| S3 | 7.253606356252422 |
| R1 | 1.2154883336883542 |
| R2 | 0.03289156611338362 |
| R3 | 5.192616584717956 |
| S1 | 0.3837561591366419 |
| S2 | 0.21091557488294085 |
| S3 | 10.440071877342897 |
| R1 | 0.37230472665443703 |
| R2 | 0.03182207311723871 |
| R3 | 3.2175053146695833 |
| S1 | 0.4925959704034994 |
| S2 | 0.701948924977828 |
| S3 | 4.90245830963505 |
| R1 | 1.3166193535450905 |
| R2 | 0.0042782984324500695 |
| R3 | 1.4269799583784553 |
| S1 | 0.7896620508680292 |
| S2 | 0.607131576764767 |
| S3 | 0.3587219290408245 |
| R1 | 0.16539187649542123 |
| R2 | 0.07030506054547442 |
| R3 | 0.20700861968829173 |
| S1 | 0.00743377030317566 |
| S2 | 0.018637209578977163 |
| S3 | 0.014256948336681669 |
| R1 | 0.0007673426522238207 |
| R2 | 0.010839434748416879 |
| R3 | 0.000967410942944915 |Alomy007222
### Chart
| Category | |
|---|---|
| S1 | 0.09682239052422296 |
| S2 | 0.021411565328110494 |
| S3 | 0.08503576460487539 |
| R1 | 0.022899952334460554 |
| R2 | 0.0653140856416467 |
| R3 | 0.007659193049278375 |
| S1 | 0.16873698993735256 |
| S2 | 7.228913877210817 |
| S3 | 0.051580116242652074 |
| R1 | 0.3354705541330854 |
| R2 | 0.047327259625552315 |
| R3 | 0.27065705563153797 |
| S1 | 0.263844612772692 |
| S2 | 1.6428015435724987 |
| S3 | 0.12740442883373507 |
| R1 | 1.5218635752222591 |
| R2 | 0.05994861793919647 |
| R3 | 0.048992357761225534 |
| S1 | 0.20982582230636362 |
| S2 | 5.029500310265818 |
| S3 | 0.2142317668370949 |
| R1 | 0.40329607330245926 |
| R2 | 0.20132926596717446 |
| R3 | 0.10280592593782255 |
| S1 | 1.0437598783735311 |
| S2 | 10.334008157305632 |
| S3 | 0.32042737644825575 |
| R1 | 0.28305663989624535 |
| R2 | 0.14202577476825284 |
| R3 | 0.01949418516009811 |
| S1 | 0.03479785334916152 |
| S2 | 0.06558253408883452 |
| S3 | 0.006556395415953232 |
| R1 | 0.24635498266823175 |
| R2 | 0.03013847376655825 |
| R3 | 0.008358893026361003 |Alomy018627
### Chart
| Category | |
|---|---|
| S1 | 0.0 |
| S2 | 0.0 |
| S3 | 0.5081084154146834 |
| R1 | 1.9361863457909934 |
| R2 | 0.38741797977394915 |
| R3 | 1.1758857203168367 |
| S1 | 0.0 |
| S2 | 0.0 |
| S3 | 1.2916925508698747 |
| R1 | 5.0376696232555345 |
| R2 | 1.6824904502538611 |
| R3 | 1.2080298658246171 |
| S1 | 0.0 |
| S2 | 0.0 |
| S3 | 0.500342402489978 |
| R1 | 3.2994668780449827 |
| R2 | 1.5017231183468265 |
| R3 | 2.0560097824139336 |
| S1 | 0.0 |
| S2 | 0.0 |
| S3 | 0.8243056503495166 |
| R1 | 4.985434954650206 |
| R2 | 0.9738104943900022 |
| R3 | 3.2906431561699736 |
| S1 | 0.0 |
| S2 | 0.0 |
| S3 | 1.1641956547110703 |
| R1 | 4.881957971562481 |
| R2 | 1.4961755156754322 |
| R3 | 1.6672969237201414 |
| S1 | 0.0 |
| S2 | 0.0025077179966250408 |
| S3 | 0.33132436002181626 |
| R1 | 4.44418466345905 |
| R2 | 2.000235024498163 |
| R3 | 4.158483446948334 |Alomy011735
Alomy003701
### Chart
| Category | |
|---|---|
| S1 | 0.5831866045426883 |
| S2 | 0.19371133764760065 |
| S3 | 0.8137439506159003 |
| R1 | 0.756856690666241 |
| R2 | 0.18585583503038494 |
| R3 | 0.7102093647215715 |
| S1 | 1.8001711002428944 |
| S2 | 2.0470263194581237 |
| S3 | 1.7286324778443831 |
| R1 | 1.1588269966766094 |
| R2 | 0.8565672119088394 |
| R3 | 2.1131985738039645 |
| S1 | 0.6719571400874343 |
| S2 | 0.5899459525855177 |
| S3 | 0.8139390343209113 |
| R1 | 1.548647614082897 |
| R2 | 0.4274838933142225 |
| R3 | 0.9832580343306923 |
| S1 | 1.1527638004520588 |
| S2 | 1.48788439384795 |
| S3 | 1.53803173722002 |
| R1 | 1.8168910331863908 |
| R2 | 0.4272500841396696 |
| R3 | 1.9206671468879353 |
| S1 | 1.1071479187206519 |
| S2 | 1.6653892192769637 |
| S3 | 0.9504793289978096 |
| R1 | 1.30516425448091 |
| R2 | 1.250256782758063 |
| R3 | 0.9589312278253536 |
| S1 | 1.06667826752866 |
| S2 | 1.1620646809246455 |
| S3 | 0.35641117695895524 |
| R1 | 2.076468274830221 |
| R2 | 0.9966235919028116 |
| R3 | 1.445594915447056 |Alomy000086
### Chart
| Category | |
|---|---|
| S1 | 0.2537353887470038 |
| S2 | 0.2757680308706284 |
| S3 | 0.40547962754546374 |
| R1 | 0.2977202775315932 |
| R2 | 2.288009671899825 |
| R3 | 0.4196854648870571 |
| S1 | 1.1947071587613265 |
| S2 | 1.7920035120806912 |
| S3 | 1.5699968858092166 |
| R1 | 1.2823284254186504 |
| R2 | 1.1589844540965715 |
| R3 | 2.153736529613987 |
| S1 | 0.6796225356261395 |
| S2 | 0.9031368304800808 |
| S3 | 1.373345781890437 |
| R1 | 0.9939244373285475 |
| R2 | 0.7444642161777937 |
| R3 | 0.9739221192577313 |
| S1 | 0.5952466905691277 |
| S2 | 1.663997288101708 |
| S3 | 1.2534721280087744 |
| R1 | 1.2394213802778993 |
| R2 | 1.0138792929889335 |
| R3 | 1.2997805581293063 |
| S1 | 0.8147524394583933 |
| S2 | 1.6963382106156708 |
| S3 | 1.2616694430114899 |
| R1 | 1.1032611360893354 |
| R2 | 1.3383841499907667 |
| R3 | 0.5873520535565137 |
| S1 | 0.8228528703284095 |
| S2 | 0.9339560879908904 |
| S3 | 0.6980812382023455 |
| R1 | 2.003354225442761 |
| R2 | 0.8744932019678527 |
| R3 | 1.072856498963818 |Alomy061728
### Chart
| Category | |
|---|---|
| S1 | 1.3050475157604664 |
| S2 | 0.3432852607474503 |
| S3 | 0.01873195731781785 |
| R1 | 1.0784967737207989 |
| R2 | 0.6669990497333517 |
| R3 | 0.7703272446888446 |
| S1 | 1.3564984686458128 |
| S2 | 1.4575385615046894 |
| S3 | 0.5892364805418333 |
| R1 | 0.7475355044700417 |
| R2 | 2.196444400701824 |
| R3 | 1.3940223196988455 |
| S1 | 0.3818971219560219 |
| S2 | 0.5434088583908301 |
| S3 | 0.32106326041812133 |
| R1 | 0.7925881849239446 |
| R2 | 0.6168403754412105 |
| R3 | 1.3766471339593245 |
| S1 | 0.39964035272248394 |
| S2 | 0.7827474342516506 |
| S3 | 0.14866286332694428 |
| R1 | 1.2384783624451794 |
| R2 | 0.461282400496013 |
| R3 | 1.267290772728602 |
| S1 | 2.8941170833557486 |
| S2 | 1.3174185706660075 |
| S3 | 0.6199719096297306 |
| R1 | 1.7386913671781137 |
| R2 | 2.089808556696477 |
| R3 | 0.37477643363398677 |
| S1 | 2.9602910162530516 |
| S2 | 1.0377392985467908 |
| S3 | 0.2293504125835161 |
| R1 | 1.337433950459203 |
| R2 | 1.6585898212277617 |
| R3 | 1.7754304716117382 |Alomy007172
### Chart
| Category | |
|---|---|
| S1 | 0.21587994851902326 |
| S2 | 0.01057019600090361 |
| S3 | 1.1258514721174826 |
| R1 | 1.0795797373974263 |
| R2 | 0.9581153730749913 |
| R3 | 1.4223625050050022 |
| S1 | 0.44147296127310104 |
| S2 | 0.34862509972408545 |
| S3 | 3.096237263022085 |
| R1 | 3.9183835178215425 |
| R2 | 3.2030642911403864 |
| R3 | 15.034112007141765 |
| S1 | 0.04677352822612046 |
| S2 | 0.05642862895752838 |
| S3 | 1.0062378860216452 |
| R1 | 4.029357404298017 |
| R2 | 4.190787972169217 |
| R3 | 1.7493102429820275 |
| S1 | 0.01761758820014348 |
| S2 | 0.7455153473669207 |
| S3 | 3.4301336217137854 |
| R1 | 4.799247640630723 |
| R2 | 4.222496357249189 |
| R3 | 7.382253920862653 |
| S1 | 0.4134034731057397 |
| S2 | 0.21184863291369616 |
| S3 | 2.686727649507649 |
| R1 | 5.077323886137942 |
| R2 | 15.784220445191735 |
| R3 | 0.7803424259166166 |
| S1 | 0.09497653854282921 |
| S2 | 0.12650197782066158 |
| S3 | 0.019538738234195708 |
| R1 | 5.791829859997198 |
| R2 | 1.64583134452599 |
| R3 | 0.23915528948274875 |Alomy049563
Alomy043265
### Chart
| Category | |
|---|---|
| S1 | 0.0 |
| S2 | 0.0 |
| S3 | 0.7242751787127458 |
| R1 | 0.28080682247327177 |
| R2 | 0.5944063655597959 |
| R3 | 0.8036734024019433 |
| S1 | 0.0 |
| S2 | 0.0 |
| S3 | 1.9621034472233507 |
| R1 | 2.7092664355242775 |
| R2 | 2.4778686135073897 |
| R3 | 4.494154492915994 |
| S1 | 0.0 |
| S2 | 0.0 |
| S3 | 1.347877454823559 |
| R1 | 4.058002744018683 |
| R2 | 2.0126060930166587 |
| R3 | 1.920031232357536 |
| S1 | 0.008032669429956306 |
| S2 | 0.0 |
| S3 | 5.270986696374869 |
| R1 | 3.762292220315315 |
| R2 | 5.293112085956926 |
| R3 | 3.7227688394305303 |
| S1 | 0.002160367031018247 |
| S2 | 0.0 |
| S3 | 2.6495479044125623 |
| R1 | 3.562020677868363 |
| R2 | 3.6465564871776013 |
| R3 | 1.069503095628452 |
| S1 | 0.0 |
| S2 | 0.0032227509835971615 |
| S3 | 0.8390047986361916 |
| R1 | 5.1765317532970085 |
| R2 | 4.0499118440801745 |
| R3 | 0.8392457929761127 |
### Chart
| Category | |
|---|---|
| S1 | 0.0 |
| S2 | 0.0 |
| S3 | 0.022363792770389523 |
| R1 | 0.49087807892627683 |
| R2 | 0.6371447617010252 |
| R3 | 1.1801992586294763 |
| S1 | 0.00291449675659018 |
| S2 | 0.0005346624817212498 |
| S3 | 0.6562866553382771 |
| R1 | 1.3710267729179482 |
| R2 | 3.3131648059907057 |
| R3 | 5.528428838254288 |
| S1 | 0.0 |
| S2 | 0.0033316257483249254 |
| S3 | 0.27986232836022124 |
| R1 | 1.9993255080321857 |
| R2 | 3.1937418081149302 |
| R3 | 3.0273237574903074 |
| S1 | 0.0 |
| S2 | 0.011524243949986046 |
| S3 | 0.46679328674527326 |
| R1 | 3.5414737883938727 |
| R2 | 4.009580721156294 |
| R3 | 7.074182976918202 |
| S1 | 0.0 |
| S2 | 0.0 |
| S3 | 0.9457549876614849 |
| R1 | 3.3212320629019345 |
| R2 | 7.676921929965612 |
| R3 | 1.9386760702449146 |
| S1 | 0.008957977843232774 |
| S2 | 0.0 |
| S3 | 0.2789718102947364 |
| R1 | 3.50388983484205 |
| R2 | 7.167007603187258 |
| R3 | 9.26759500149763 |
### Chart
| Category | |
|---|---|
| S1 | 0.0008081105538368984 |
| S2 | 0.013812522825087313 |
| S3 | 0.03963110378136989 |
| R1 | 0.4432285794811744 |
| R2 | 0.4356689514671577 |
| R3 | 0.6920909949379744 |
| S1 | 0.023791193960537086 |
| S2 | 0.036039198040707286 |
| S3 | 0.4908757029490127 |
| R1 | 1.4147662451740806 |
| R2 | 7.466412856251494 |
| R3 | 6.811221378752063 |
| S1 | 0.008867791123097124 |
| S2 | 0.009764590727187829 |
| S3 | 0.29329195910645184 |
| R1 | 2.4164413854545064 |
| R2 | 3.0309115862080396 |
| R3 | 7.188514871693922 |
| S1 | 0.034914389764727743 |
| S2 | 0.08462527784627456 |
| S3 | 0.7478956894313068 |
| R1 | 1.2254538943247857 |
| R2 | 10.832723512831132 |
| R3 | 15.028685495361799 |
| S1 | 0.060570359452269885 |
| S2 | 0.01470495014897504 |
| S3 | 0.7462363832176689 |
| R1 | 2.3939220475673797 |
| R2 | 11.966731632756295 |
| R3 | 2.2448667043551596 |
| S1 | 0.01143897942714847 |
| S2 | 0.021040856860224732 |
| S3 | 0.5397115566779499 |
| R1 | 3.8661814312498515 |
| R2 | 16.59012569543454 |
| R3 | 19.306227999927927 |Alomy013820
### Chart
| Category | |
|---|---|
| S1 | 0.0 |
| S2 | 0.16305639708762154 |
| S3 | 0.023097900839557487 |
| R1 | 1.0984673217169716 |
| R2 | 0.8439241587688819 |
| R3 | 0.00013812293342143679 |
| S1 | 0.0005713551235812067 |
| S2 | 2.881388498320819 |
| S3 | 2.424057476437025 |
| R1 | 1.8528820436214002 |
| R2 | 6.669172917827506 |
| R3 | 0.002500782473483867 |
| S1 | 0.0 |
| S2 | 1.2963570471784147 |
| S3 | 0.7983863587466344 |
| R1 | 2.8657395993305315 |
| R2 | 2.4137789781657393 |
| R3 | 0.0 |
| S1 | 0.00021839889989021572 |
| S2 | 7.124625659117409 |
| S3 | 1.4070012652382544 |
| R1 | 8.616376019504527 |
| R2 | 8.386615022192048 |
| R3 | 0.0 |
| S1 | 0.0009505310014264069 |
| S2 | 4.253304387504545 |
| S3 | 3.4128407005651433 |
| R1 | 9.62474178952826 |
| R2 | 11.642966295496628 |
| R3 | 0.0 |
| S1 | 0.0 |
| S2 | 8.362894590217978 |
| S3 | 1.82777346371769 |
| R1 | 21.016256394892526 |
| R2 | 28.746780474682467 |
| R3 | 0.0020858954783384773 |Alomy025188
### Chart
| Category | |
|---|---|
| S1 | 0.024783975334820574 |
| S2 | 0.007650538055686297 |
| S3 | 0.17695785237661923 |
| R1 | 0.04977458443931707 |
| R2 | 0.9429832496582157 |
| R3 | 0.47838815171371474 |
| S1 | 0.0727852305956662 |
| S2 | 0.0998337857773738 |
| S3 | 0.1904133625568479 |
| R1 | 0.2272600193929044 |
| R2 | 0.48475745279168764 |
| R3 | 0.9323631386860597 |
| S1 | 0.0066237361715974765 |
| S2 | 0.006812788113164243 |
| S3 | 0.07209780409358389 |
| R1 | 0.46410812308223565 |
| R2 | 0.25075635478927893 |
| R3 | 0.22720578126031932 |
| S1 | 0.05381673637729375 |
| S2 | 1.04781302194457 |
| S3 | 1.237375674406094 |
| R1 | 1.14555573102447 |
| R2 | 6.705222006187916 |
| R3 | 4.008419792085658 |
| S1 | 0.00876746352433621 |
| S2 | 0.0467255591974502 |
| S3 | 0.06145445459471072 |
| R1 | 0.16814861747093293 |
| R2 | 0.40694323611224864 |
| R3 | 0.46348703495542587 |
| S1 | 0.15774139764598907 |
| S2 | 0.38371616988442475 |
| S3 | 0.14337472290427478 |
| R1 | 2.714102850793147 |
| R2 | 3.615259601863189 |
| R3 | 1.2040393962290623 |Alomy011081
### Chart
| Category | |
|---|---|
| S1 | 0.0 |
| S2 | 0.9399336466551222 |
| S3 | 4.06833907192241 |
| R1 | 0.0 |
| R2 | 0.0017370578474831663 |
| R3 | 0.0004772847953628229 |
| S1 | 0.0004231627157878565 |
| S2 | 5.340762560501264 |
| S3 | 5.274758738585304 |
| R1 | 0.00010173954006139313 |
| R2 | 0.0 |
| R3 | 0.0006013124384872921 |
| S1 | 0.0 |
| S2 | 3.71428558297398 |
| S3 | 2.5073085062812495 |
| R1 | 0.0 |
| R2 | 0.00017045281360987186 |
| R3 | 0.0 |
| S1 | 0.0 |
| S2 | 8.617104714671727 |
| S3 | 8.854571488824343 |
| R1 | 0.0011144308783881099 |
| R2 | 0.004398997052285882 |
| R3 | 0.0037486025063652507 |
| S1 | 0.007460355788946239 |
| S2 | 9.045768941719755 |
| S3 | 6.517976479664678 |
| R1 | 0.00110557079025548 |
| R2 | 0.0018578636585307635 |
| R3 | 0.00012676797883215494 |
| S1 | 0.001483472842149959 |
| S2 | 4.427294738195136 |
| S3 | 3.2031981763642725 |
| R1 | 0.0 |
| R2 | 0.0 |
| R3 | 0.00046950495662007415 |Alomy000024
### Chart
| Category | |
|---|---|
| S1 | 4.505646095242255 |
| S2 | 3.074101967095224 |
| S3 | 5.652677308692731 |
| R1 | 0.006296610329445471 |
| R2 | 5.484524448338109 |
| R3 | 1.19570121111753 |
| S1 | 7.467129215325578 |
| S2 | 16.45883351838897 |
| S3 | 11.104119419163991 |
| R1 | 0.06808228304370349 |
| R2 | 7.810485670205324 |
| R3 | 0.22485169698082064 |
| S1 | 4.4345142783682086 |
| S2 | 11.627728307718106 |
| S3 | 5.071659409146887 |
| R1 | 0.04515636341948673 |
| R2 | 15.533888150597015 |
| R3 | 0.2085595876289044 |
| S1 | 6.314454655998595 |
| S2 | 20.116304892422246 |
| S3 | 13.511050055684679 |
| R1 | 0.13125820643132974 |
| R2 | 13.485779573593659 |
| R3 | 0.7055525278295093 |
| S1 | 10.358970187137665 |
| S2 | 20.703557725094985 |
| S3 | 9.15084193658417 |
| R1 | 0.0419222541230638 |
| R2 | 19.39386890070831 |
| R3 | 0.14184874924584234 |
| S1 | 7.5264761021790285 |
| S2 | 8.028727685251154 |
| S3 | 5.645608935275778 |
| R1 | 0.00794986663177843 |
| R2 | 9.06059339160027 |
| R3 | 6.584774039168038 |Alomy003479
### Chart
| Category | |
|---|---|
| S1 | 0.1785531718546566 |
| S2 | 0.1716670234602603 |
| S3 | 0.046529517092796756 |
| R1 | 0.5867957515388241 |
| R2 | 0.3482051967932978 |
| R3 | 0.4480064667166401 |
| S1 | 1.4147156009118387 |
| S2 | 0.29451106129813315 |
| S3 | 0.004971115362502863 |
| R1 | 0.11164806982969865 |
| R2 | 3.0135118827746066 |
| R3 | 0.6625809942901834 |
| S1 | 0.13200091092012997 |
| S2 | 0.7181717562044367 |
| S3 | 0.03875641994442169 |
| R1 | 4.298530424108826 |
| R2 | 0.49342230250412616 |
| R3 | 1.4427971220575924 |
| S1 | 0.034405078547570335 |
| S2 | 0.31294316502186115 |
| S3 | 0.01605515118446629 |
| R1 | 4.533998211956649 |
| R2 | 0.37643353465467994 |
| R3 | 2.3539969818212136 |
| S1 | 0.14617215393776858 |
| S2 | 0.006214689276698999 |
| S3 | 0.0015975254034350402 |
| R1 | 0.36252057019852213 |
| R2 | 0.2913810373598558 |
| R3 | 0.5603283132053687 |
| S1 | 0.0016852780406532647 |
| S2 | 0.05844694564983211 |
| S3 | 0.01001424500184458 |
| R1 | 0.08129871141707391 |
| R2 | 0.7768094629988441 |
| R3 | 0.05012259736156998 |Alomy036282
### Chart
| Category | |
|---|---|
| S1 | 0.7398389773949215 |
| S2 | 0.5727585797238987 |
| S3 | 0.13170155233171366 |
| R1 | 0.5370878519822048 |
| R2 | 1.0065706979671125 |
| R3 | 0.5506739576136567 |
| S1 | 2.2397974224358523 |
| S2 | 6.160426308421738 |
| S3 | 2.537745498378175 |
| R1 | 1.085358513391591 |
| R2 | 3.180102321178419 |
| R3 | 3.6700125050942467 |
| S1 | 0.8314455654106386 |
| S2 | 0.9876426808334248 |
| S3 | 0.5185889857902738 |
| R1 | 4.753304510200935 |
| R2 | 1.1265250158470534 |
| R3 | 3.3213188915169796 |
| S1 | 1.8246416991775192 |
| S2 | 3.4466474467348633 |
| S3 | 1.03638229262572 |
| R1 | 6.734324111786816 |
| R2 | 4.503499169347833 |
| R3 | 6.285656971278303 |
| S1 | 5.628491492936017 |
| S2 | 1.672491420104585 |
| S3 | 0.822812328074703 |
| R1 | 2.668523749148083 |
| R2 | 2.281367813883618 |
| R3 | 1.0035716577536522 |
| S1 | 12.009353792830415 |
| S2 | 3.9299923072071543 |
| S3 | 1.4346520143978883 |
| R1 | 12.316558159220873 |
| R2 | 5.480686298348952 |
| R3 | 10.73673175548909 |Alomy010160
Figure S4: RT-qPCR expression patterns of the 21 contigs used for RNA-Seq expression data validation. The expression values were measured in each of the three resistant F2 plants (R1, R2, R3; red bars) and each of the three sensitive F2 plants (S1, S2, S3; green bars) used for RNA-Seq in each experimental modality. RT-qPCR expression data is normalised using three reference genes.
