# Supplementary material for: ALOMYbase, a resource to investigate non-target-site-based resistance to herbicides inhibiting acetolactate-synthase (ALS) in the major grass weed Alopecurus myosuroides (black-grass)
Source: BMC Genomics. 2015 Aug 12;16(1):590. doi: 10.1186/s12864-015-1804-x (PMC4534104; doi:10.1186/s12864-015-1804-x)
Supplement: Additional file 5: Figure S5. — RT-qPCR validation of the RNA-Seq expression patterns of 21 contigs. The expression values were computed for the resistant (R, black bars) or the sensitive pool (S, white bars) for each experimental modality. Normalised expression values were measured by RT-qPCR and averaged for the F2 three plants in each pool (A) or were computed as RPKM values from RNA-Seq data (B). Pearson’s coefficient correlation computed between RT-qPCR and RNA-Seq expression patterns are given in red. (PPTX 148 kb) [file 12864_2015_1804_MOESM5_ESM.pptx]

## Slide 1
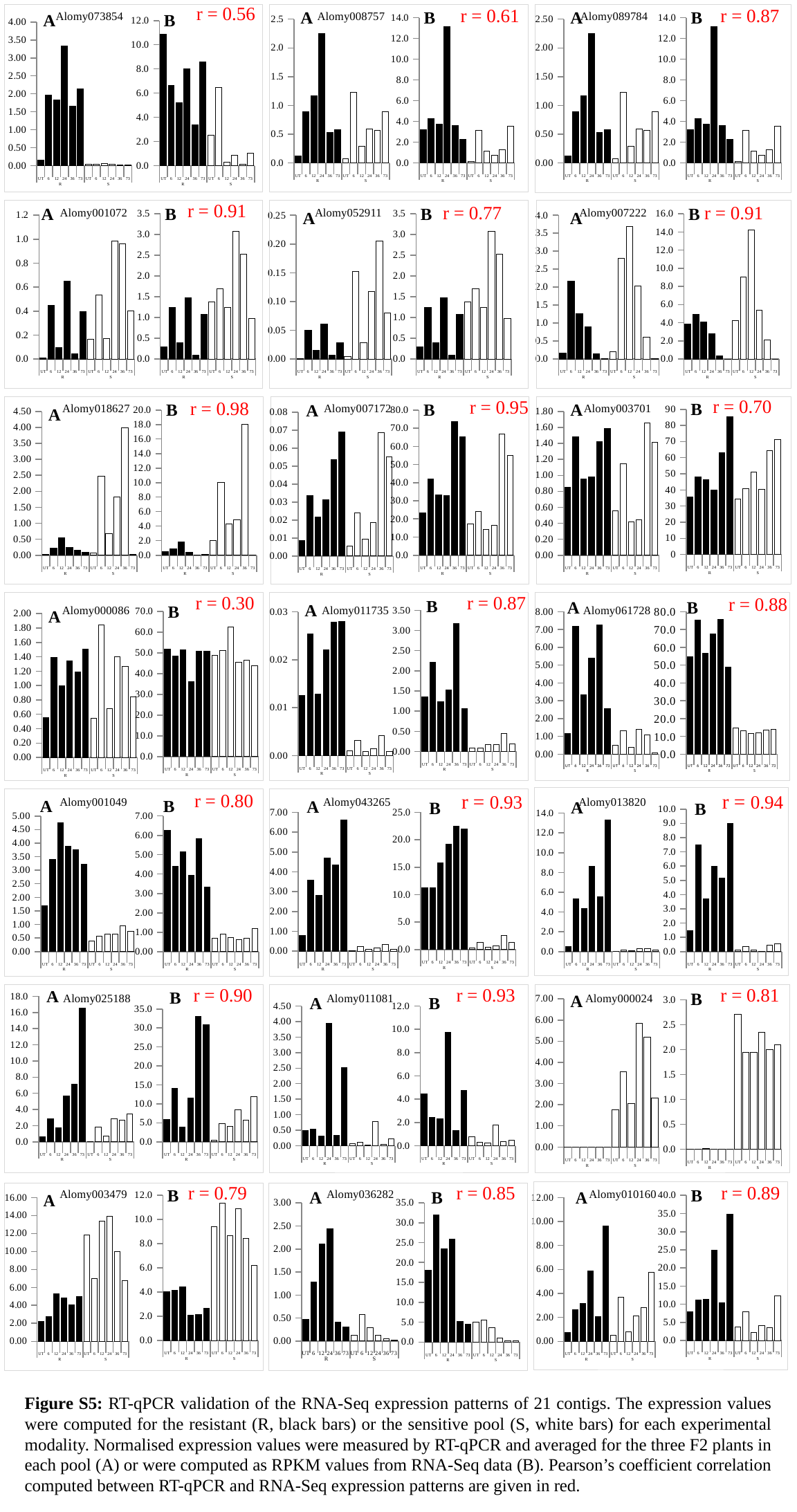

| r = 0.56 |
| --- |
| r = 0.61 |
| --- |
| r = 0.87 |
| --- |
Alomy073854
### Chart: A
| Category | qRT-PCR |
|---|---|
| UT | 0.119669599 |
| 6 | 0.885731247 |
| 12 | 1.161528623 |
| 24 | 2.249364641 |
| 36 | 0.53516961 |
| 73 | 0.579773384 |
| UT | 0.076241645 |
| 6 | 1.23311161 |
| 12 | 0.29297961 |
| 24 | 0.590210151 |
| 36 | 0.561864243 |
| 73 | 0.890500757 |
### Chart: B
| Category | RPKM |
|---|---|
| UT | 3.18 |
| 6 | 4.3 |
| 12 | 3.71 |
| 24 | 13.14 |
| 36 | 3.62 |
| 73 | 2.26 |
| UT | 0.13 |
| 6 | 3.16 |
| 12 | 1.12 |
| 24 | 0.74 |
| 36 | 1.26 |
| 73 | 3.53 |
Alomy008757
### Chart: A
| Category | qRT-PCR |
|---|---|
| UT | 0.119669599 |
| 6 | 0.885731247 |
| 12 | 1.161528623 |
| 24 | 2.249364641 |
| 36 | 0.53516961 |
| 73 | 0.579773384 |
| UT | 0.076241645 |
| 6 | 1.23311161 |
| 12 | 0.29297961 |
| 24 | 0.590210151 |
| 36 | 0.561864243 |
| 73 | 0.890500757 |
### Chart: B
| Category | RPKM |
|---|---|
| UT | 3.18 |
| 6 | 4.3 |
| 12 | 3.71 |
| 24 | 13.14 |
| 36 | 3.62 |
| 73 | 2.26 |
| UT | 0.13 |
| 6 | 3.16 |
| 12 | 1.12 |
| 24 | 0.74 |
| 36 | 1.26 |
| 73 | 3.53 |Alomy089784
### Chart: A
| Category | qRT-PCR |
|---|---|
| UT | 0.15875491750352944 |
| 6 | 1.960232088141326 |
| 12 | 1.8338843033586094 |
| 24 | 3.3392210138679954 |
| 36 | 1.6557266759730276 |
| 73 | 2.1447225613136083 |
| UT | 0.048496967480040676 |
| 6 | 0.04237240868806802 |
| 12 | 0.06544903250761121 |
| 24 | 0.036591659295747196 |
| 36 | 0.013804778451714706 |
| 73 | 0.013688654427557366 |
### Chart: B
| Category | RPKM |
|---|---|
| UT | 10.89 |
| 6 | 6.64 |
| 12 | 5.2 |
| 24 | 7.99 |
| 36 | 3.37 |
| 73 | 8.61 |
| UT | 2.53 |
| 6 | 6.45 |
| 12 | 0.29 |
| 24 | 0.87 |
| 36 | 0.1 |
| 73 | 1.05 || r = 0.91 |
| --- |
| r = 0.77 |
| --- |
| r = 0.91 |
| --- |
### Chart: A
| Category | qRT-PCR |
|---|---|
| UT | 0.006407506768365836 |
| 6 | 0.4506133040709046 |
| 12 | 0.09775089375433511 |
| 24 | 0.6520074543131581 |
| 36 | 0.04514531283111023 |
| 73 | 0.3943617995073206 |
| UT | 0.16587210163699018 |
| 6 | 0.5351805690453294 |
| 12 | 0.17356585844771488 |
| 24 | 0.986046758242814 |
| 36 | 0.9637555930537544 |
| 73 | 0.4038680280044012 |
### Chart: B
| Category | RPKM |
|---|---|
| UT | 0.3 |
| 6 | 1.24 |
| 12 | 0.39 |
| 24 | 1.47 |
| 36 | 0.09 |
| 73 | 1.07 |
| UT | 1.37 |
| 6 | 1.7 |
| 12 | 1.24 |
| 24 | 3.08 |
| 36 | 2.53 |
| 73 | 0.98 |
### Chart: A
| Category | qRT-PCR |
|---|---|
| UT | 0.00021812204809953795 |
| 6 | 0.0495221072981659 |
| 12 | 0.015201151201023977 |
| 24 | 0.06101463420400139 |
| 36 | 0.006410758407889977 |
| 73 | 0.028742339064892777 |
| UT | 0.003784111069301016 |
| 6 | 0.15284871756517895 |
| 12 | 0.028292957623405706 |
| 24 | 0.11716070157862786 |
| 36 | 0.20491219449171236 |
| 73 | 0.07975162364382603 |
### Chart: B
| Category | RPKM |
|---|---|
| UT | 0.3 |
| 6 | 1.24 |
| 12 | 0.39 |
| 24 | 1.47 |
| 36 | 0.09 |
| 73 | 1.07 |
| UT | 1.37 |
| 6 | 1.7 |
| 12 | 1.24 |
| 24 | 3.08 |
| 36 | 2.53 |
| 73 | 0.98 |
### Chart: A
| Category | qRT-PCR |
|---|---|
| UT | 0.1645231407397524 |
| 6 | 2.1623571292057626 |
| 12 | 1.2598859512204719 |
| 24 | 0.891239126713642 |
| 36 | 0.14795632662084235 |
| 73 | 0.004205974217340188 |
| UT | 0.2029102579890718 |
| 6 | 2.7936434340857397 |
| 12 | 3.693378399388383 |
| 24 | 2.0335441392720717 |
| 36 | 0.599269000555134 |
| 73 | 0.013796117562307293 |
### Chart: B
| Category | RPKM |
|---|---|
| UT | 3.86 |
| 6 | 4.93 |
| 12 | 4.08 |
| 24 | 2.8 |
| 36 | 0.39 |
| 73 | 0.0 |
| UT | 4.22 |
| 6 | 9.02 |
| 12 | 14.19 |
| 24 | 5.39 |
| 36 | 2.11 |
| 73 | 0.0 |
Alomy001072
Alomy052911
Alomy007222
| r = 0.98 |
| --- |
| r = 0.95 |
| --- |
| r = 0.70 |
| --- |
### Chart: B
| Category | RPKM |
|---|---|
| UT | 35.47 |
| 6 | 47.91 |
| 12 | 46.53 |
| 24 | 39.74 |
| 36 | 62.93 |
| 73 | 85.5 |
| UT | 34.21 |
| 6 | 40.62 |
| 12 | 51.27 |
| 24 | 40.33 |
| 36 | 64.42 |
| 73 | 71.13 |
### Chart: A
| Category | qRT-PCR |
|---|---|
| UT | 0.8532300181193743 |
| 6 | 1.4800171716404205 |
| 12 | 0.9573020650158087 |
| 24 | 0.9789525947373575 |
| 36 | 1.4219902087000953 |
| 73 | 1.5895763030347352 |
| UT | 0.5579230712783695 |
| 6 | 1.1443379907578337 |
| 12 | 0.4169910477242736 |
| 24 | 0.4457974482229539 |
| 36 | 1.6549289105727683 |
| 73 | 1.415245830154084 |
### Chart: A
| Category | qRT-PCR |
|---|---|
| UT | 0.03215254733696085 |
| 6 | 0.21882722562224854 |
| 12 | 0.545883581811969 |
| 24 | 0.23307730472562493 |
| 36 | 0.14959256936776652 |
| 73 | 0.09440499756074162 |
| UT | 0.07006696158422034 |
| 6 | 2.476424310519421 |
| 12 | 0.6732014340724731 |
| 24 | 1.8313132686190674 |
| 36 | 3.986918020842525 |
| 73 | 0.03540271193554368 |
### Chart: B
| Category | RPKM |
|---|---|
| UT | 0.49 |
| 6 | 0.83 |
| 12 | 1.83 |
| 24 | 0.4 |
| 36 | 0.0 |
| 73 | 0.09 |
| UT | 1.99 |
| 6 | 10.01 |
| 12 | 4.28 |
| 24 | 4.9 |
| 36 | 18.02 |
| 73 | 0.06 |Alomy018627
### Chart: B
| Category | RPKM |
|---|---|
| UT | 23.49 |
| 6 | 42.11 |
| 12 | 33.11 |
| 24 | 32.98 |
| 36 | 73.47 |
| 73 | 65.41 |
| UT | 17.25 |
| 6 | 24.07 |
| 12 | 14.08 |
| 24 | 16.3 |
| 36 | 66.85 |
| 73 | 54.8 |
### Chart: A
| Category | qRT-PCR |
|---|---|
| UT | 0.008586366272841891 |
| 6 | 0.03344136538604895 |
| 12 | 0.021486007298032444 |
| 24 | 0.03130512731149793 |
| 36 | 0.053599476193388305 |
| 73 | 0.06888171347479026 |
| UT | 0.005602770926392016 |
| 6 | 0.02409166966875394 |
| 12 | 0.009245306525068978 |
| 24 | 0.018477079380924504 |
| 36 | 0.06853385828435421 |
| 73 | 0.05523064398827652 |
Alomy007172
Alomy003701
| r = 0.30 |
| --- |
| r = 0.87 |
| --- |
| r = 0.88 |
| --- |
### Chart: B
| Category | RPKM |
|---|---|
| UT | 1.35 |
| 6 | 2.21 |
| 12 | 1.24 |
| 24 | 1.53 |
| 36 | 3.18 |
| 73 | 1.06 |
| UT | 0.08 |
| 6 | 0.08 |
| 12 | 0.17 |
| 24 | 0.17 |
| 36 | 0.44 |
| 73 | 0.19 |
### Chart: A
| Category | qRT-PCR |
|---|---|
| UT | 0.012503320894514522 |
| 6 | 0.025407866186772782 |
| 12 | 0.012812199847151836 |
| 24 | 0.02207675746952494 |
| 36 | 0.027892949835707743 |
| 73 | 0.027956027537584305 |
| UT | 0.0010425202975360055 |
| 6 | 0.0032109858778615794 |
| 12 | 0.0008891087491065264 |
| 24 | 0.0014638413364688556 |
| 36 | 0.0041742813773453236 |
| 73 | 0.0009102835041024567 |
### Chart: A
| Category | qRT-PCR |
|---|---|
| UT | 1.146782151305587 |
| 6 | 7.199853966510976 |
| 12 | 3.34890359965931 |
| 24 | 5.3834274367630215 |
| 36 | 7.259370418326138 |
| 73 | 2.551015984994803 |
| UT | 0.49355909075586535 |
| 6 | 1.3152369161186497 |
| 12 | 0.3775067339287174 |
| 24 | 1.4058815291891094 |
| 36 | 1.0747309032891448 |
| 73 | 0.08287335737115312 |
### Chart: B
| Category | RPKM |
|---|---|
| UT | 54.64 |
| 6 | 75.38 |
| 12 | 56.61 |
| 24 | 67.4 |
| 36 | 75.7 |
| 73 | 48.74 |
| UT | 14.64 |
| 6 | 13.18 |
| 12 | 11.58 |
| 24 | 11.93 |
| 36 | 13.75 |
| 73 | 13.96 |Alomy000086
Alomy011735
Alomy061728
### Chart: B
| Category | RPKM |
|---|---|
| UT | 51.76 |
| 6 | 48.66 |
| 12 | 51.55 |
| 24 | 35.99 |
| 36 | 50.87 |
| 73 | 50.66 |
| UT | 48.9 |
| 6 | 51.17 |
| 12 | 62.62 |
| 24 | 45.39 |
| 36 | 46.6 |
| 73 | 43.75 |
### Chart: A
| Category | qRT-PCR |
|---|---|
| UT | 0.5557287291479854 |
| 6 | 1.3877060119318951 |
| 12 | 1.0003342112417897 |
| 24 | 1.3455230794551991 |
| 36 | 1.1848467068616806 |
| 73 | 1.509470789819608 |
| UT | 0.5427267244223762 |
| 6 | 1.8458143789426265 |
| 12 | 0.6813445576376692 |
| 24 | 1.403058524508879 |
| 36 | 1.2619784713822877 |
| 73 | 0.840135396414096 |
| r = 0.80 |
| --- |
| r = 0.93 |
| --- |
| r = 0.94 |
| --- |
Alomy001049
Alomy043265
Alomy013820
### Chart: A
| Category | qRT-PCR |
|---|---|
| UT | 1.6944972233486546 |
| 6 | 3.3864355346546007 |
| 12 | 4.746870053224058 |
| 24 | 3.880721112690272 |
| 36 | 3.74575874373091 |
| 73 | 3.2100342262256345 |
| UT | 0.39557318105856076 |
| 6 | 0.576004522586219 |
| 12 | 0.635740104439022 |
| 24 | 0.6569178154634577 |
| 36 | 0.9392923893291328 |
| 73 | 0.7512145093535899 |
### Chart: B
| Category | RPKM |
|---|---|
| UT | 6.26 |
| 6 | 4.4 |
| 12 | 5.16 |
| 24 | 3.95 |
| 36 | 5.83 |
| 73 | 3.34 |
| UT | 0.68 |
| 6 | 0.89 |
| 12 | 0.72 |
| 24 | 0.62 |
| 36 | 0.7 |
| 73 | 1.19 |
### Chart: A
| Category | qRT-PCR |
|---|---|
| UT | 0.7799376186597572 |
| 6 | 3.5798677759178332 |
| 12 | 2.7989677844237346 |
| 24 | 4.704404932965249 |
| 36 | 4.345094041786074 |
| 73 | 6.611860433396921 |
| UT | 0.006725798720365272 |
| 6 | 0.21879860735495948 |
| 12 | 0.09192288112983273 |
| 24 | 0.15645480076077237 |
| 36 | 0.3180189176822778 |
| 73 | 0.0879328747491915 |
### Chart: B
| Category | RPKM |
|---|---|
| UT | 11.27 |
| 6 | 11.28 |
| 12 | 15.76 |
| 24 | 19.16 |
| 36 | 22.42 |
| 73 | 22.0 |
| UT | 0.28 |
| 6 | 1.24 |
| 12 | 0.38 |
| 24 | 0.63 |
| 36 | 2.58 |
| 73 | 1.33 |
### Chart: A
| Category | qRT-PCR |
|---|---|
| UT | 0.5340483733113989 |
| 6 | 5.31476185964515 |
| 12 | 4.339646785205808 |
| 24 | 8.640571185523482 |
| 36 | 5.551546370233017 |
| 73 | 13.29918183058531 |
| UT | 0.016433100256316613 |
| 6 | 0.18364827681563867 |
| 12 | 0.10452100216681552 |
| 24 | 0.288338005188489 |
| 36 | 0.26976890408394255 |
| 73 | 0.16901989337188658 |
### Chart: B
| Category | RPKM |
|---|---|
| UT | 1.47 |
| 6 | 7.47 |
| 12 | 3.7 |
| 24 | 5.99 |
| 36 | 5.15 |
| 73 | 8.99 |
| UT | 0.09 |
| 6 | 0.33 |
| 12 | 0.11 |
| 24 | 0.02 |
| 36 | 0.44 |
| 73 | 0.54 || r = 0.90 |
| --- |
| r = 0.93 |
| --- |
| r = 0.81 |
| --- |
### Chart: A
| Category | qRT-PCR |
|---|---|
| UT | 0.6465367572379292 |
| 6 | 2.8683325130233808 |
| 12 | 1.7633981358899484 |
| 24 | 5.65870600237957 |
| 36 | 7.168362585878917 |
| 73 | 16.555462234843404 |
| UT | 0.06172692336109189 |
| 6 | 1.791349048835543 |
| 12 | 0.7087117019678825 |
| 24 | 2.849524668389073 |
| 36 | 2.656294452242529 |
| 73 | 3.409823999168553 |
### Chart: B
| Category | RPKM |
|---|---|
| UT | 5.93 |
| 6 | 14.1 |
| 12 | 3.91 |
| 24 | 11.59 |
| 36 | 33.07 |
| 73 | 30.89 |
| UT | 0.44 |
| 6 | 4.82 |
| 12 | 4.04 |
| 24 | 8.41 |
| 36 | 5.76 |
| 73 | 11.98 |
### Chart: A
| Category | qRT-PCR |
|---|---|
| UT | 0.0007980745073585807 |
| 6 | 0.0002976787783634892 |
| 12 | 5.8206005058115315e-05 |
| 24 | 0.003577221368608162 |
| 36 | 0.0010762582045138905 |
| 73 | 0.00017847054328380644 |
| UT | 1.7442995873692475 |
| 6 | 3.55887274657424 |
| 12 | 2.048195961888734 |
| 24 | 5.833642618364439 |
| 36 | 5.206202248726943 |
| 73 | 2.326996377237568 |
### Chart: B
| Category | RPKM |
|---|---|
| UT | 0.0 |
| 6 | 0.0 |
| 12 | 0.01 |
| 24 | 0.0 |
| 36 | 0.0 |
| 73 | 0.0 |
| UT | 2.71 |
| 6 | 1.94 |
| 12 | 1.95 |
| 24 | 2.35 |
| 36 | 2.0 |
| 73 | 2.1 |Alomy025188
Alomy011081
Alomy000024
### Chart: A
| Category | qRT-PCR |
|---|---|
| UT | 0.5012142463980095 |
| 6 | 0.5423109715157214 |
| 12 | 0.3155372529142967 |
| 24 | 3.9374683708938023 |
| 36 | 0.34538978674926946 |
| 73 | 2.5216765479285637 |
| UT | 0.07202191131034152 |
| 6 | 0.12250776981661733 |
| 12 | 0.03145734303837123 |
| 24 | 0.7792626159663798 |
| 36 | 0.03973287701870686 |
| 73 | 0.22190900039225592 |
### Chart: B
| Category | RPKM |
|---|---|
| UT | 4.44 |
| 6 | 2.41 |
| 12 | 2.29 |
| 24 | 9.77 |
| 36 | 1.33 |
| 73 | 4.78 |
| UT | 0.75 |
| 6 | 0.32 |
| 12 | 0.23 |
| 24 | 1.76 |
| 36 | 0.38 |
| 73 | 0.48 || r = 0.79 |
| --- |
| r = 0.85 |
| --- |
| r = 0.89 |
| --- |
Alomy003479
Alomy036282
Alomy010160
### Chart: B
| Category | RPKM |
|---|---|
| UT | 4.05 |
| 6 | 4.14 |
| 12 | 4.45 |
| 24 | 2.11 |
| 36 | 2.15 |
| 73 | 2.68 |
| UT | 9.43 |
| 6 | 11.33 |
| 12 | 8.68 |
| 24 | 10.88 |
| 36 | 8.42 |
| 73 | 6.19 |
### Chart: B
| Category | RPKM |
|---|---|
| UT | 7.97 |
| 6 | 11.09 |
| 12 | 11.42 |
| 24 | 24.82 |
| 36 | 10.5 |
| 73 | 34.85 |
| UT | 3.76 |
| 6 | 7.88 |
| 12 | 2.13 |
| 24 | 4.06 |
| 36 | 3.56 |
| 73 | 12.32 |
### Chart: A
| Category | qRT-PCR |
|---|---|
| UT | 2.2283615118843696 |
| 6 | 2.735365009707454 |
| 12 | 5.285262337399305 |
| 24 | 4.844694510024637 |
| 36 | 4.064475765882089 |
| 73 | 5.0041217423748146 |
| UT | 11.85220152431404 |
| 6 | 7.007832925082042 |
| 12 | 13.33987712344132 |
| 24 | 13.908858259408253 |
| 36 | 9.981773952010641 |
| 73 | 6.732410547221154 |
### Chart: A
| Category | qRT-PCR |
|---|---|
| UT | 0.7101308015138467 |
| 6 | 2.622892819983297 |
| 12 | 3.1455623022802874 |
| 24 | 5.882398103189236 |
| 36 | 2.031985637787872 |
| 73 | 9.60148301021619 |
| UT | 0.4902821803266175 |
| 6 | 3.6988855265202756 |
| 12 | 0.7814755208468914 |
| 24 | 2.1094240804764586 |
| 36 | 2.8049004977054017 |
| 73 | 5.767332996674507 |
### Chart: A
| Category | qRT-PCR |
|---|---|
| UT | 0.469306134794701 |
| 6 | 1.2770448413183848 |
| 12 | 2.1105035895503783 |
| 24 | 2.4377596411776143 |
| 36 | 0.4079643272314975 |
| 73 | 0.30359221332055575 |
| UT | 0.13368478229057912 |
| 6 | 0.5784928773567485 |
| 12 | 0.29740033529796706 |
| 24 | 0.12119944788479524 |
| 36 | 0.05234651328971537 |
| 73 | 0.02384529383465203 |
### Chart: B
| Category | RPKM |
|---|---|
| UT | 18.01 |
| 6 | 31.94 |
| 12 | 23.36 |
| 24 | 25.85 |
| 36 | 5.1 |
| 73 | 4.52 |
| UT | 5.01 |
| 6 | 5.48 |
| 12 | 3.63 |
| 24 | 1.03 |
| 36 | 0.38 |
| 73 | 0.27 |Figure S5: RT-qPCR validation of the RNA-Seq expression patterns of 21 contigs. The expression values were computed for the resistant (R, black bars) or the sensitive pool (S, white bars) for each experimental modality. Normalised expression values were measured by RT-qPCR and averaged for the three F2 plants in each pool (A) or were computed as RPKM values from RNA-Seq data (B). Pearson’s coefficient correlation computed between RT-qPCR and RNA-Seq expression patterns are given in red.
