# Supplementary material for: ALOMYbase, a resource to investigate non-target-site-based resistance to herbicides inhibiting acetolactate-synthase (ALS) in the major grass weed Alopecurus myosuroides (black-grass)
Source: BMC Genomics. 2015 Aug 12;16(1):590. doi: 10.1186/s12864-015-1804-x (PMC4534104; doi:10.1186/s12864-015-1804-x)
Supplement: Additional file 6: Figure S6. — Number of contigs differentially regulated between phenotypes. Six-way Venn diagrams show the number of contigs up-regulated at each time-point in the sensitive pool compared to the resistant pool (A) or in the resistant pool compared to the sensitive pool (B). UT, untreated; xHAT, x hours after herbicide treatment. (PPTX 111 kb) [file 12864_2015_1804_MOESM6_ESM.pptx]

## Slide 1
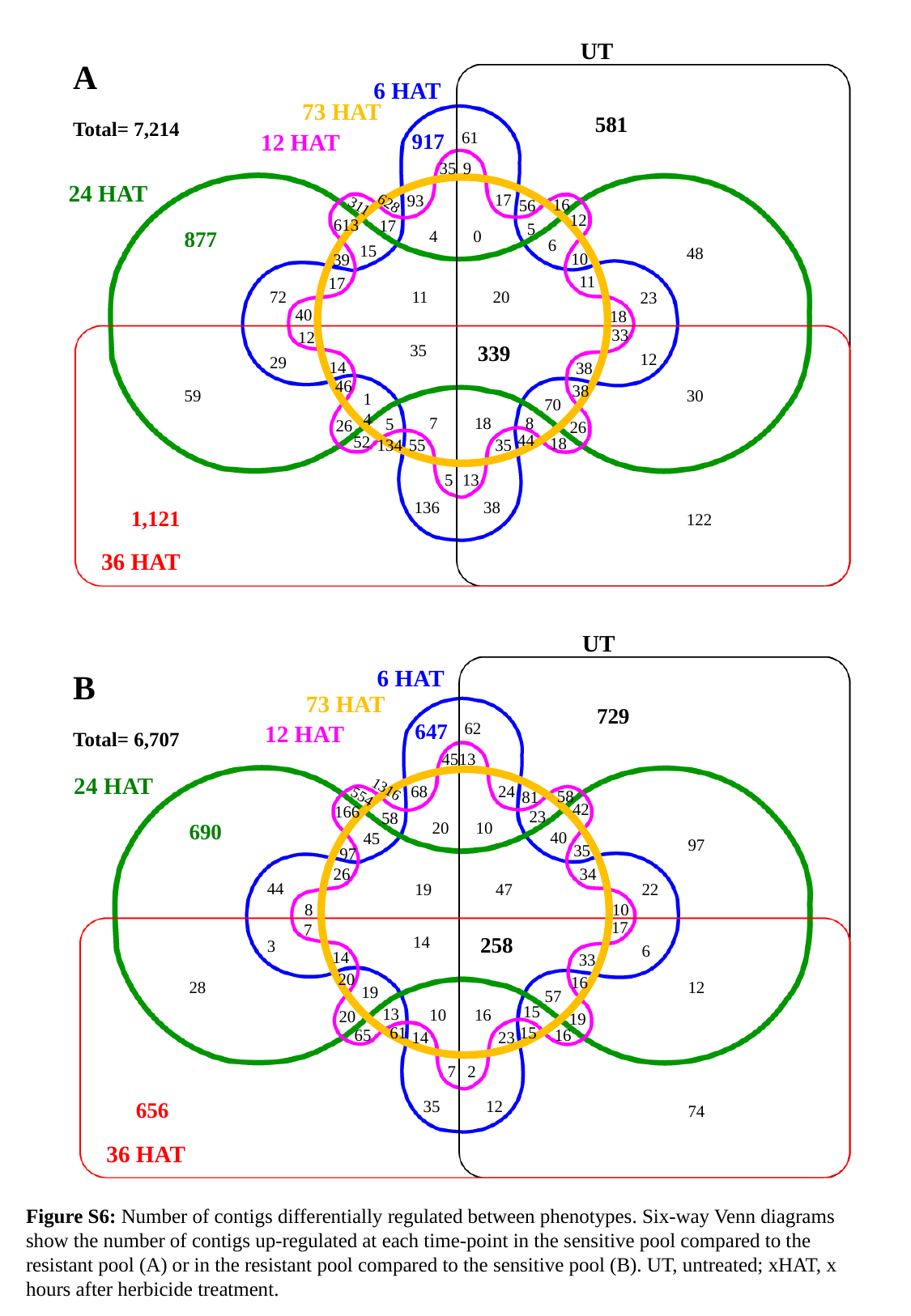

UT
A
Total= 7,214
6 HAT
73 HAT
581
917
61
12 HAT
35
9
24 HAT
17
93
16
56
628
311
12
613
17
5
4
0
877
6
15
48
10
39
11
17
72
11
20
23
40
18
33
12
35
339
12
29
14
38
46
38
59
30
14
70
8
7
18
5
26
26
44
52
18
55
35
134
5
13
136
38
1,121
122
36 HAT
UT
6 HAT
73 HAT
729
647
62
12 HAT
45
13
24 HAT
1316
68
24
58
81
554
42
166
23
58
20
10
690
40
45
97
35
97
26
34
44
19
47
22
10
8
17
7
14
258
3
6
14
33
20
16
28
12
19
57
15
13
10
16
20
19
61
15
65
16
14
23
7
2
35
12
656
74
36 HAT
B
Total= 6,707
Figure S6: Number of contigs differentially regulated between phenotypes. Six-way Venn diagrams show the number of contigs up-regulated at each time-point in the sensitive pool compared to the resistant pool (A) or in the resistant pool compared to the sensitive pool (B). UT, untreated; xHAT, x hours after herbicide treatment.
