# Supplementary material for: ALOMYbase, a resource to investigate non-target-site-based resistance to herbicides inhibiting acetolactate-synthase (ALS) in the major grass weed Alopecurus myosuroides (black-grass)
Source: BMC Genomics. 2015 Aug 12;16(1):590. doi: 10.1186/s12864-015-1804-x (PMC4534104; doi:10.1186/s12864-015-1804-x)
Supplement: Additional file 7: Figure S7. — RT-qPCR expression patterns of 11 candidate NTSR contigs. The expression values were measured in each of the three resistant F2 plants (R1, R2, R3; red bars) and each of the three sensitive F2 plants (S1, S2, S3; green bars) in each experimental modality used for RNA-Seq. RT-qPCR expression data is normalised using three reference genes. CYP, cytochrome P450; Perox, peroxidase; GT, glycosyltransferase; HeLo, helix-loop-helix DNA-binding protein; DP, disease resistance protein. Figure S8. RT-qPCR validation of the RNA-Seq expression patterns of the 11 candidate NTSR contigs. The expression values were computed for the resistant (R, black bars) or the sensitive pool (S, white bars) for each experimental modality. Normalised expression values were measured by RT-qPCR and averaged for the three F2 plants in each pool (A) or were computed as RPKM values from RNA-Seq data (B). Pearson’s coefficient correlation computed between RT-qPCR and RNA-Seq expression patterns are given in red. CYP, cytochrome P450; Perox, peroxidase; GT, glycosyltransferase; HeLo, helix-loop-helix DNA-binding protein; DP, disease resistance protein. Figure S9. Individual relative expression levels (log10) measured by RT-qPCR of the five candidate NTSR contigs showing a higher expression in resistant plants plotted by increasing value for 35 A. myosuroides F2 plants. Green, sensitive plants; red, resistant plants. (*) indicate the three resistant and the three sensitive F2 plants used for RNA-Seq. (PPTX 1295 kb) [file 12864_2015_1804_MOESM7_ESM.pptx]

## Slide 1
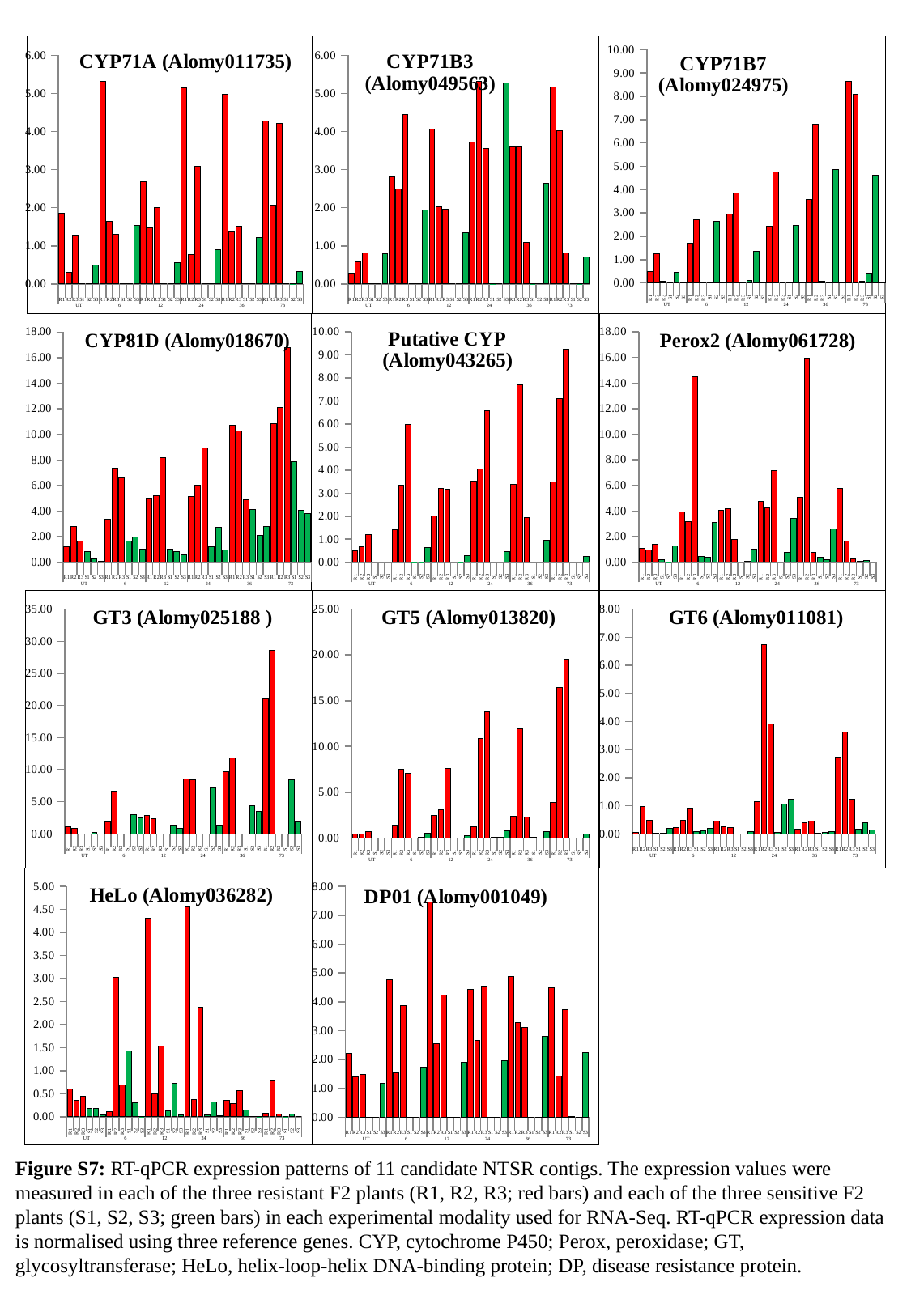

### Chart: CYP71A (Alomy011735)
| Category | |
|---|---|
| R1 | 1.8524921508161534 |
| R2 | 0.299250642925155 |
| R3 | 1.290576091577888 |
| S1 | 0.0 |
| S2 | 0.0 |
| S3 | 0.49199762324677654 |
| R1 | 5.31031314471803 |
| R2 | 1.634632273652115 |
| R3 | 1.2954621995743945 |
| S1 | 0.0 |
| S2 | 0.0 |
| S3 | 1.5385975036083281 |
| R1 | 2.687379164377664 |
| R2 | 1.4699025839993474 |
| R3 | 2.0124746948928536 |
| S1 | 0.0 |
| S2 | 0.0 |
| S3 | 0.5589847092501169 |
| R1 | 5.148028257400653 |
| R2 | 0.781384356180966 |
| R3 | 3.0937712269956794 |
| S1 | 0.0 |
| S2 | 0.0 |
| S3 | 0.8921277650710681 |
| R1 | 4.979796695221102 |
| R2 | 1.357714523555026 |
| R3 | 1.5163844244201798 |
| S1 | 0.0 |
| S2 | 0.0 |
| S3 | 1.2180296321680224 |
| R1 | 4.272455768115927 |
| R2 | 2.0591056923872406 |
| R3 | 4.222573036107523 |
| S1 | 0.0 |
| S2 | 0.0024575281911770838 |
| S3 | 0.3198811096221484 |
### Chart: CYP71B3 (Alomy049563)
| Category | |
|---|---|
| R1 | 0.28763574749378246 |
| R2 | 0.5765075914735006 |
| R3 | 0.8056315842544177 |
| S1 | 0.0 |
| S2 | 0.0 |
| S3 | 0.7839530883723541 |
| R1 | 2.816451049812694 |
| R2 | 2.494298701706171 |
| R3 | 4.449145656320365 |
| S1 | 0.0 |
| S2 | 0.0 |
| S3 | 1.9429967644010884 |
| R1 | 4.05919798929643 |
| R2 | 2.017973509146594 |
| R3 | 1.9613672664531028 |
| S1 | 0.0 |
| S2 | 0.0 |
| S3 | 1.3428395989329003 |
| R1 | 3.732957903646577 |
| R2 | 5.320706762643719 |
| R3 | 3.562186366388759 |
| S1 | 0.007203098946862986 |
| S2 | 0.0 |
| S3 | 5.276786665085483 |
| R1 | 3.5872493855743572 |
| R2 | 3.599183449207347 |
| R3 | 1.081873518296392 |
| S1 | 0.002563836193687967 |
| S2 | 0.0 |
| S3 | 2.651235973632539 |
| R1 | 5.172901658534721 |
| R2 | 4.028467364441298 |
| R3 | 0.8048985012626821 |
| S1 | 0.0 |
| S2 | 0.003225261709909923 |
| S3 | 0.7068128648043245 |
### Chart: CYP71B7 (Alomy024975)
| Category | |
|---|---|
| R1 | 0.5042323459178253 |
| R2 | 1.2515112172075602 |
| R3 | 0.06316071774825374 |
| S1 | 0.020050574101832794 |
| S2 | 0.47496719156957645 |
| S3 | 0.016876208801807742 |
| R1 | 1.7125633696931652 |
| R2 | 2.7198285276985703 |
| R3 | 0.01731205175733942 |
| S1 | 0.012167656293308531 |
| S2 | 2.647595609724711 |
| S3 | 0.05052691857957319 |
| R1 | 2.956908708833412 |
| R2 | 3.858937589079314 |
| R3 | 0.01103243344270887 |
| S1 | 0.1153992772761562 |
| S2 | 1.3621993807570965 |
| S3 | 0.008335002156344546 |
| R1 | 2.443527376644523 |
| R2 | 4.770864105403275 |
| R3 | 0.04818976880574763 |
| S1 | 0.028074242858270496 |
| S2 | 2.476457443092129 |
| S3 | 0.026115071616875526 |
| R1 | 3.5857488494111145 |
| R2 | 6.79885890921884 |
| R3 | 0.062125990621803046 |
| S1 | 0.03387243160631801 |
| S2 | 4.8648815577772755 |
| S3 | 0.046747976965404364 |
| R1 | 8.62498509335396 |
| R2 | 8.090968812868745 |
| R3 | 0.06597256629619402 |
| S1 | 0.4114379285072496 |
| S2 | 4.6259526201732415 |
| S3 | 0.028286052710280687 |
### Chart: CYP81D (Alomy018670)
| Category | |
|---|---|
| R1 | 1.1948782800903786 |
| R2 | 2.7737645600222525 |
| R3 | 1.653019113755606 |
| S1 | 0.8521026245713142 |
| S2 | 0.2922859944246339 |
| S3 | 0.0770772838571919 |
| R1 | 3.342330300127723 |
| R2 | 7.341919869030263 |
| R3 | 6.6410313195854105 |
| S1 | 1.6399152315898455 |
| S2 | 1.9767944385296394 |
| S3 | 1.0408532396183539 |
| R1 | 5.032432011427143 |
| R2 | 5.1863507797744095 |
| R3 | 8.149671456313104 |
| S1 | 1.0034215842051648 |
| S2 | 0.8526675785838937 |
| S3 | 0.5569939321458832 |
| R1 | 5.149547062990432 |
| R2 | 6.024115910818276 |
| R3 | 8.917373004126912 |
| S1 | 1.1927025521607186 |
| S2 | 2.712651555734248 |
| S3 | 0.9497844113109599 |
| R1 | 10.728334256331333 |
| R2 | 10.299802749256266 |
| R3 | 4.9079790362864655 |
| S1 | 4.144711195148782 |
| S2 | 2.086963830554444 |
| S3 | 2.77305094314442 |
| R1 | 10.804787443811112 |
| R2 | 12.078527195990734 |
| R3 | 16.811461077985662 |
| S1 | 7.86943093960503 |
| S2 | 4.071340541252347 |
| S3 | 3.7871145135401685 |
### Chart: Putative CYP (Alomy043265)
| Category | |
|---|---|
| R1 | 0.48821171647053185 |
| R2 | 0.6629561576374837 |
| R3 | 1.188644981871256 |
| S1 | 0.0 |
| S2 | 0.0 |
| S3 | 0.020177396161095814 |
| R1 | 1.4016068265945578 |
| R2 | 3.3477488668577413 |
| R3 | 5.990247634301199 |
| S1 | 0.003108826552896593 |
| S2 | 0.0006155923463771916 |
| S3 | 0.6526714031656047 |
| R1 | 2.0036091031105108 |
| R2 | 3.2073869639070614 |
| R3 | 3.1859072862536317 |
| S1 | 0.0 |
| S2 | 0.003538339049291713 |
| S3 | 0.27223030434020645 |
| R1 | 3.511716114973859 |
| R2 | 4.030296629956457 |
| R3 | 6.57120205396543 |
| S1 | 0.0 |
| S2 | 0.011530754058402325 |
| S3 | 0.4578336482239148 |
| R1 | 3.363047292464005 |
| R2 | 7.718493347795843 |
| R3 | 1.953741485098373 |
| S1 | 0.0 |
| S2 | 0.0 |
| S3 | 0.9540567530468334 |
| R1 | 3.4897734644018312 |
| R2 | 7.102657335244631 |
| R3 | 9.243150500544303 |
| S1 | 0.008317790299503055 |
| S2 | 0.0 |
| S3 | 0.2554808339480715 |
### Chart: Perox2 (Alomy061728)
| Category | |
|---|---|
| R1 | 1.075245188542584 |
| R2 | 0.9683819590085105 |
| R3 | 1.3967193063656655 |
| S1 | 0.20919732756168952 |
| S2 | 0.011802345576848214 |
| S3 | 1.2596775991290583 |
| R1 | 3.937485892671627 |
| R2 | 3.1926266282088456 |
| R3 | 14.469449378652456 |
| S1 | 0.44891553786685834 |
| S2 | 0.36227402333835207 |
| S3 | 3.1345211871507384 |
| R1 | 4.036002340323817 |
| R2 | 4.207957174540777 |
| R3 | 1.802751284113337 |
| S1 | 0.044585430169905405 |
| S2 | 0.0599297869632574 |
| S3 | 1.0280049846529895 |
| R1 | 4.755791928799245 |
| R2 | 4.244140917034974 |
| R3 | 7.150349464454846 |
| S1 | 0.015798139350471137 |
| S2 | 0.7623664056047206 |
| S3 | 3.4394800426121366 |
| R1 | 5.049253079629333 |
| R2 | 15.935672108306797 |
| R3 | 0.7931860670422851 |
| S1 | 0.3933435638023175 |
| S2 | 0.21267579282405066 |
| S3 | 2.6181733532410663 |
| R1 | 5.759876050843215 |
| R2 | 1.6478291954925053 |
| R3 | 0.2453427086486879 |
| S1 | 0.10194562534521746 |
| S2 | 0.12693378534564426 |
| S3 | 0.019740661422597644 |
### Chart: GT3 (Alomy025188 )
| Category | |
|---|---|
| R1 | 1.089697484153943 |
| R2 | 0.8497706757194594 |
| R3 | 0.00014211184038522907 |
| S1 | 0.0 |
| S2 | 0.16349252955439372 |
| S3 | 0.02168824052888193 |
| R1 | 1.8908356466434704 |
| R2 | 6.712028648387706 |
| R3 | 0.0021332440389655793 |
| S1 | 0.0005458169809344839 |
| S2 | 2.9345218119383496 |
| S3 | 2.438979517587346 |
| R1 | 2.8726413033682263 |
| R2 | 2.417553104301619 |
| R3 | 0.0 |
| S1 | 0.0 |
| S2 | 1.301038348481066 |
| S3 | 0.8250967574225816 |
| R1 | 8.546853482886839 |
| R2 | 8.42926452425187 |
| R3 | 0.0 |
| S1 | 0.00021923017718859057 |
| S2 | 7.135480282282186 |
| S3 | 1.4128744927078438 |
| R1 | 9.72688020379795 |
| R2 | 11.7782075538388 |
| R3 | 0.0 |
| S1 | 0.0011280517382876627 |
| S2 | 4.434182457006497 |
| S3 | 3.533572847982803 |
| R1 | 21.082271781593843 |
| R2 | 28.582257639869965 |
| R3 | 0.0018572830664000232 |
| S1 | 0.0 |
| S2 | 8.358879379824128 |
| S3 | 1.870592617681531 |
### Chart: GT5 (Alomy013820)
| Category | |
|---|---|
| R1 | 0.44075648814738555 |
| R2 | 0.4432886248144266 |
| R3 | 0.7181000069723844 |
| S1 | 0.0007830952777079198 |
| S2 | 0.01527988452803781 |
| S3 | 0.03323632096320411 |
| R1 | 1.4124192618425364 |
| R2 | 7.4807417428745335 |
| R3 | 7.051124574218379 |
| S1 | 0.02472623399319313 |
| S2 | 0.03837410206664481 |
| S3 | 0.48784449438707805 |
| R1 | 2.422740471990325 |
| R2 | 3.0438219928836747 |
| R3 | 7.552377890743422 |
| S1 | 0.009328645106696322 |
| S2 | 0.009275793395168049 |
| S3 | 0.29495856799858217 |
| R1 | 1.2129353628840225 |
| R2 | 10.886408464964273 |
| R3 | 13.822369728722148 |
| S1 | 0.03698295984456001 |
| S2 | 0.08375303051487991 |
| S3 | 0.744278025206027 |
| R1 | 2.4218432038234523 |
| R2 | 11.967988255476527 |
| R3 | 2.264807651399071 |
| S1 | 0.06020414853369962 |
| S2 | 0.015335727703823722 |
| S3 | 0.7337668360143041 |
| R1 | 3.8772033203774092 |
| R2 | 16.48039695536265 |
| R3 | 19.539945216015866 |
| S1 | 0.012510619024170576 |
| S2 | 0.02114707283907904 |
| S3 | 0.47340198825241003 |
### Chart: GT6 (Alomy011081)
| Category | |
|---|---|
| R1 | 0.05255576978387451 |
| R2 | 0.9709892067317509 |
| R3 | 0.4800977626784031 |
| S1 | 0.025653554544881274 |
| S2 | 0.008469257598746775 |
| S3 | 0.18194292178739654 |
| R1 | 0.23063908730918914 |
| R2 | 0.4917015503659544 |
| R3 | 0.9045922768720209 |
| S1 | 0.07483748080563912 |
| S2 | 0.10141549494875998 |
| S3 | 0.19127033369545293 |
| R1 | 0.4638220612081197 |
| R2 | 0.2517906321782962 |
| R3 | 0.23099906535647402 |
| S1 | 0.006611783826275717 |
| S2 | 0.006510687089511437 |
| S3 | 0.08124955819932653 |
| R1 | 1.1462315006439394 |
| R2 | 6.74102702814856 |
| R3 | 3.925146583888907 |
| S1 | 0.05943825043664687 |
| S2 | 1.0535236434310282 |
| S3 | 1.2248259540314643 |
| R1 | 0.16778693354393032 |
| R2 | 0.4033808117402887 |
| R3 | 0.46500161496358927 |
| S1 | 0.010139339061671346 |
| S2 | 0.04412663585040816 |
| S3 | 0.06493265614404108 |
| R1 | 2.7268997510138764 |
| R2 | 3.6094026674252917 |
| R3 | 1.2287272253465233 |
| S1 | 0.15168176884158902 |
| S2 | 0.3834733969460622 |
| S3 | 0.13057183538911651 |
### Chart: HeLo (Alomy036282)
| Category | |
|---|---|
| R1 | 0.6030273554259461 |
| R2 | 0.35082458313745596 |
| R3 | 0.4540664658207011 |
| S1 | 0.17915613280994835 |
| S2 | 0.17321678756040915 |
| S3 | 0.04868142650137987 |
| R1 | 0.11329037284619077 |
| R2 | 3.020177294638375 |
| R3 | 0.6976668564705886 |
| S1 | 1.4281737338413696 |
| S2 | 0.30221416545992585 |
| S3 | 0.005090732768950127 |
| R1 | 4.305360295800834 |
| R2 | 0.4968451591532471 |
| R3 | 1.5293053136970531 |
| S1 | 0.13138810649487997 |
| S2 | 0.7226761640865598 |
| S3 | 0.03813673531246124 |
| R1 | 4.55012786000548 |
| R2 | 0.3783506617368262 |
| R3 | 2.384800401790536 |
| S1 | 0.034248012333968375 |
| S2 | 0.31384771015821855 |
| S3 | 0.015502621162198766 |
| R1 | 0.363436090432305 |
| R2 | 0.2947812980713612 |
| R3 | 0.5656755931908263 |
| S1 | 0.14908852369527872 |
| S2 | 0.006228426613117047 |
| S3 | 0.001722589560750323 |
| R1 | 0.08189734357633859 |
| R2 | 0.7770852382674145 |
| R3 | 0.051794058117914266 |
| S1 | 0.0018571053273439434 |
| S2 | 0.05847013051894956 |
| S3 | 0.011208645657662601 |
### Chart: DP01 (Alomy001049)
| Category | |
|---|---|
| R1 | 2.2048904119643566 |
| R2 | 1.4038258555298295 |
| R3 | 1.4747754025517779 |
| S1 | 0.0028566049352594138 |
| S2 | 0.0 |
| S3 | 1.1838629382404229 |
| R1 | 4.761131577202136 |
| R2 | 1.5320162632410308 |
| R3 | 3.8661587635206365 |
| S1 | 0.0027413848665157484 |
| S2 | 0.001470540766828294 |
| S3 | 1.7238016421253133 |
| R1 | 7.455991291833627 |
| R2 | 2.559639018860081 |
| R3 | 4.224979848978465 |
| S1 | 0.000341262750069869 |
| S2 | 0.003899202772106772 |
| S3 | 1.9029798477948892 |
| R1 | 4.4326873014708585 |
| R2 | 2.6600129204873495 |
| R3 | 4.549463116112606 |
| S1 | 0.00048797014726440017 |
| S2 | 0.00472881728263863 |
| S3 | 1.9655366589604704 |
| R1 | 4.864925742543534 |
| R2 | 3.2657532594776217 |
| R3 | 3.1065972291715744 |
| S1 | 0.0037362630463032686 |
| S2 | 0.002148220417223803 |
| S3 | 2.8119926845238714 |
| R1 | 4.4814442445766804 |
| R2 | 1.4320276384931696 |
| R3 | 3.716630795607055 |
| S1 | 0.015521448753620957 |
| S2 | 0.00043229911382505016 |
| S3 | 2.2376897801933238 |Figure S7: RT-qPCR expression patterns of 11 candidate NTSR contigs. The expression values were measured in each of the three resistant F2 plants (R1, R2, R3; red bars) and each of the three sensitive F2 plants (S1, S2, S3; green bars) in each experimental modality used for RNA-Seq. RT-qPCR expression data is normalised using three reference genes. CYP, cytochrome P450; Perox, peroxidase; GT, glycosyltransferase; HeLo, helix-loop-helix DNA-binding protein; DP, disease resistance protein.

## Slide 2
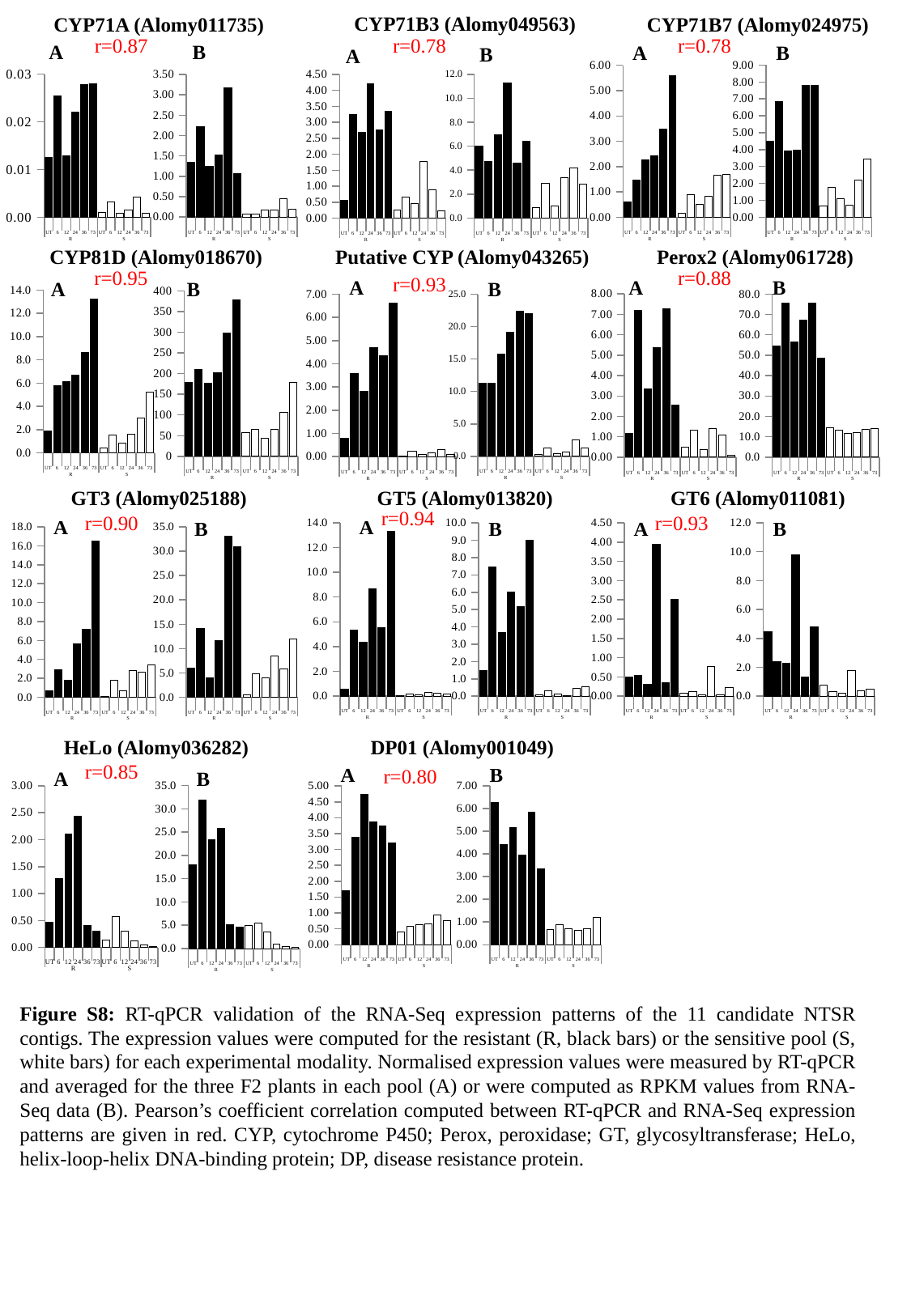

CYP71B3 (Alomy049563)
CYP71A (Alomy011735)
CYP71B7 (Alomy024975)
r=0.87
r=0.78
r=0.78
### Chart: A
| Category | qRT-PCR |
|---|---|
| UT | 0.6063014269578797 |
| 6 | 1.4832346497163584 |
| 12 | 2.2756262437851453 |
| 24 | 2.420860416951182 |
| 36 | 3.482244583083919 |
| 73 | 5.593975490839632 |
| UT | 0.17063132482440568 |
| 6 | 0.9034300615325311 |
| 12 | 0.4953112200631991 |
| 24 | 0.8435489191890917 |
| 36 | 1.6485006554496657 |
| 73 | 1.6885588671302576 |
### Chart: B
| Category | RPKM |
|---|---|
| UT | 4.5 |
| 6 | 6.85 |
| 12 | 3.94 |
| 24 | 3.96 |
| 36 | 7.79 |
| 73 | 7.82 |
| UT | 0.68 |
| 6 | 1.78 |
| 12 | 1.11 |
| 24 | 0.69 |
| 36 | 2.18 |
| 73 | 3.45 |
### Chart: A
| Category | qRT-PCR |
|---|---|
| UT | 0.5565916410739002 |
| 6 | 3.253298469279743 |
| 12 | 2.6795129216320426 |
| 24 | 4.205283677559685 |
| 36 | 2.756102117692699 |
| 73 | 3.335422508079567 |
| UT | 0.26131769612411804 |
| 6 | 0.6476655881336961 |
| 12 | 0.4476131996443001 |
| 24 | 1.761329921344115 |
| 36 | 0.8845999366087423 |
| 73 | 0.2366793755047448 |
### Chart: B
| Category | RPKM |
|---|---|
| UT | 6.03 |
| 6 | 4.72 |
| 12 | 6.97 |
| 24 | 11.24 |
| 36 | 4.61 |
| 73 | 6.39 |
| UT | 0.89 |
| 6 | 2.87 |
| 12 | 1.04 |
| 24 | 3.36 |
| 36 | 4.2 |
| 73 | 2.83 |
### Chart: A
| Category | qRT-PCR |
|---|---|
| UT | 0.012503320894514522 |
| 6 | 0.025407866186772782 |
| 12 | 0.012812199847151836 |
| 24 | 0.02207675746952494 |
| 36 | 0.027892949835707743 |
| 73 | 0.027956027537584305 |
| UT | 0.0010425202975360055 |
| 6 | 0.0032109858778615794 |
| 12 | 0.0008891087491065264 |
| 24 | 0.0014638413364688556 |
| 36 | 0.0041742813773453236 |
| 73 | 0.0009102835041024567 |
### Chart: B
| Category | RPKM |
|---|---|
| UT | 1.35 |
| 6 | 2.21 |
| 12 | 1.24 |
| 24 | 1.53 |
| 36 | 3.18 |
| 73 | 1.06 |
| UT | 0.08 |
| 6 | 0.08 |
| 12 | 0.17 |
| 24 | 0.17 |
| 36 | 0.44 |
| 73 | 0.19 |CYP81D (Alomy018670)
Putative CYP (Alomy043265)
Perox2 (Alomy061728)
r=0.88
r=0.95
r=0.93
### Chart: A
| Category | qRT-PCR |
|---|---|
| UT | 0.7799376186597572 |
| 6 | 3.5798677759178332 |
| 12 | 2.7989677844237346 |
| 24 | 4.704404932965249 |
| 36 | 4.345094041786074 |
| 73 | 6.611860433396921 |
| UT | 0.006725798720365272 |
| 6 | 0.21879860735495948 |
| 12 | 0.09192288112983273 |
| 24 | 0.15645480076077237 |
| 36 | 0.3180189176822778 |
| 73 | 0.0879328747491915 |
### Chart: B
| Category | RPKM |
|---|---|
| UT | 11.27 |
| 6 | 11.28 |
| 12 | 15.76 |
| 24 | 19.16 |
| 36 | 22.42 |
| 73 | 22.0 |
| UT | 0.28 |
| 6 | 1.24 |
| 12 | 0.38 |
| 24 | 0.63 |
| 36 | 2.58 |
| 73 | 1.33 |
### Chart: A
| Category | qRT-PCR |
|---|---|
| UT | 1.146782151305587 |
| 6 | 7.199853966510976 |
| 12 | 3.34890359965931 |
| 24 | 5.3834274367630215 |
| 36 | 7.259370418326138 |
| 73 | 2.551015984994803 |
| UT | 0.49355909075586535 |
| 6 | 1.3152369161186497 |
| 12 | 0.3775067339287174 |
| 24 | 1.4058815291891094 |
| 36 | 1.0747309032891448 |
| 73 | 0.08287335737115312 |
### Chart: B
| Category | RPKM |
|---|---|
| UT | 54.64 |
| 6 | 75.38 |
| 12 | 56.61 |
| 24 | 67.4 |
| 36 | 75.7 |
| 73 | 48.74 |
| UT | 14.64 |
| 6 | 13.18 |
| 12 | 11.58 |
| 24 | 11.93 |
| 36 | 13.75 |
| 73 | 13.96 |
### Chart: A
| Category | qRT-PCR |
|---|---|
| UT | 1.8738873179560789 |
| 6 | 5.775093829581132 |
| 12 | 6.122818082504885 |
| 24 | 6.697011992645206 |
| 36 | 8.645372013958022 |
| 73 | 13.23159190592917 |
| UT | 0.40715530095104663 |
| 6 | 1.5525209699126128 |
| 12 | 0.8043610316449805 |
| 24 | 1.6183795064019755 |
| 36 | 3.001575322949215 |
| 73 | 5.242628664799182 |
### Chart: B
| Category | RPKM |
|---|---|
| UT | 179.24 |
| 6 | 210.04 |
| 12 | 177.01 |
| 24 | 201.61 |
| 36 | 297.42 |
| 73 | 377.22 |
| UT | 56.95 |
| 6 | 64.86 |
| 12 | 43.91 |
| 24 | 65.38 |
| 36 | 106.97 |
| 73 | 178.38 |GT3 (Alomy025188)
GT5 (Alomy013820)
GT6 (Alomy011081)
r=0.94
r=0.90
r=0.93
### Chart: A
| Category | qRT-PCR |
|---|---|
| UT | 0.5340483733113989 |
| 6 | 5.31476185964515 |
| 12 | 4.339646785205808 |
| 24 | 8.640571185523482 |
| 36 | 5.551546370233017 |
| 73 | 13.29918183058531 |
| UT | 0.016433100256316613 |
| 6 | 0.18364827681563867 |
| 12 | 0.10452100216681552 |
| 24 | 0.288338005188489 |
| 36 | 0.26976890408394255 |
| 73 | 0.16901989337188658 |
### Chart: B
| Category | RPKM |
|---|---|
| UT | 1.47 |
| 6 | 7.47 |
| 12 | 3.7 |
| 24 | 5.99 |
| 36 | 5.15 |
| 73 | 8.99 |
| UT | 0.09 |
| 6 | 0.33 |
| 12 | 0.11 |
| 24 | 0.02 |
| 36 | 0.44 |
| 73 | 0.54 |
### Chart: A
| Category | qRT-PCR |
|---|---|
| UT | 0.5012142463980095 |
| 6 | 0.5423109715157214 |
| 12 | 0.3155372529142967 |
| 24 | 3.9374683708938023 |
| 36 | 0.34538978674926946 |
| 73 | 2.5216765479285637 |
| UT | 0.07202191131034152 |
| 6 | 0.12250776981661733 |
| 12 | 0.03145734303837123 |
| 24 | 0.7792626159663798 |
| 36 | 0.03973287701870686 |
| 73 | 0.22190900039225592 |
### Chart: B
| Category | RPKM |
|---|---|
| UT | 4.44 |
| 6 | 2.41 |
| 12 | 2.29 |
| 24 | 9.77 |
| 36 | 1.33 |
| 73 | 4.78 |
| UT | 0.75 |
| 6 | 0.32 |
| 12 | 0.23 |
| 24 | 1.76 |
| 36 | 0.38 |
| 73 | 0.48 |
### Chart: A
| Category | qRT-PCR |
|---|---|
| UT | 0.6465367572379292 |
| 6 | 2.8683325130233808 |
| 12 | 1.7633981358899484 |
| 24 | 5.65870600237957 |
| 36 | 7.168362585878917 |
| 73 | 16.555462234843404 |
| UT | 0.06172692336109189 |
| 6 | 1.791349048835543 |
| 12 | 0.7087117019678825 |
| 24 | 2.849524668389073 |
| 36 | 2.656294452242529 |
| 73 | 3.409823999168553 |
### Chart: B
| Category | RPKM |
|---|---|
| UT | 5.93 |
| 6 | 14.1 |
| 12 | 3.91 |
| 24 | 11.59 |
| 36 | 33.07 |
| 73 | 30.89 |
| UT | 0.44 |
| 6 | 4.82 |
| 12 | 4.04 |
| 24 | 8.41 |
| 36 | 5.76 |
| 73 | 11.98 |HeLo (Alomy036282)
DP01 (Alomy001049)
r=0.85
r=0.80
### Chart: A
| Category | qRT-PCR |
|---|---|
| UT | 1.6944972233486546 |
| 6 | 3.3864355346546007 |
| 12 | 4.746870053224058 |
| 24 | 3.880721112690272 |
| 36 | 3.74575874373091 |
| 73 | 3.2100342262256345 |
| UT | 0.39557318105856076 |
| 6 | 0.576004522586219 |
| 12 | 0.635740104439022 |
| 24 | 0.6569178154634577 |
| 36 | 0.9392923893291328 |
| 73 | 0.7512145093535899 |
### Chart: B
| Category | RPKM |
|---|---|
| UT | 6.26 |
| 6 | 4.4 |
| 12 | 5.16 |
| 24 | 3.95 |
| 36 | 5.83 |
| 73 | 3.34 |
| UT | 0.68 |
| 6 | 0.89 |
| 12 | 0.72 |
| 24 | 0.62 |
| 36 | 0.7 |
| 73 | 1.19 |
### Chart: A
| Category | qRT-PCR |
|---|---|
| UT | 0.469306134794701 |
| 6 | 1.2770448413183848 |
| 12 | 2.1105035895503783 |
| 24 | 2.4377596411776143 |
| 36 | 0.4079643272314975 |
| 73 | 0.30359221332055575 |
| UT | 0.13368478229057912 |
| 6 | 0.5784928773567485 |
| 12 | 0.29740033529796706 |
| 24 | 0.12119944788479524 |
| 36 | 0.05234651328971537 |
| 73 | 0.02384529383465203 |
### Chart: B
| Category | RPKM |
|---|---|
| UT | 18.01 |
| 6 | 31.94 |
| 12 | 23.36 |
| 24 | 25.85 |
| 36 | 5.1 |
| 73 | 4.52 |
| UT | 5.01 |
| 6 | 5.48 |
| 12 | 3.63 |
| 24 | 1.03 |
| 36 | 0.38 |
| 73 | 0.27 |Figure S8: RT-qPCR validation of the RNA-Seq expression patterns of the 11 candidate NTSR contigs. The expression values were computed for the resistant (R, black bars) or the sensitive pool (S, white bars) for each experimental modality. Normalised expression values were measured by RT-qPCR and averaged for the three F2 plants in each pool (A) or were computed as RPKM values from RNA-Seq data (B). Pearson’s coefficient correlation computed between RT-qPCR and RNA-Seq expression patterns are given in red. CYP, cytochrome P450; Perox, peroxidase; GT, glycosyltransferase; HeLo, helix-loop-helix DNA-binding protein; DP, disease resistance protein.

## Slide 3
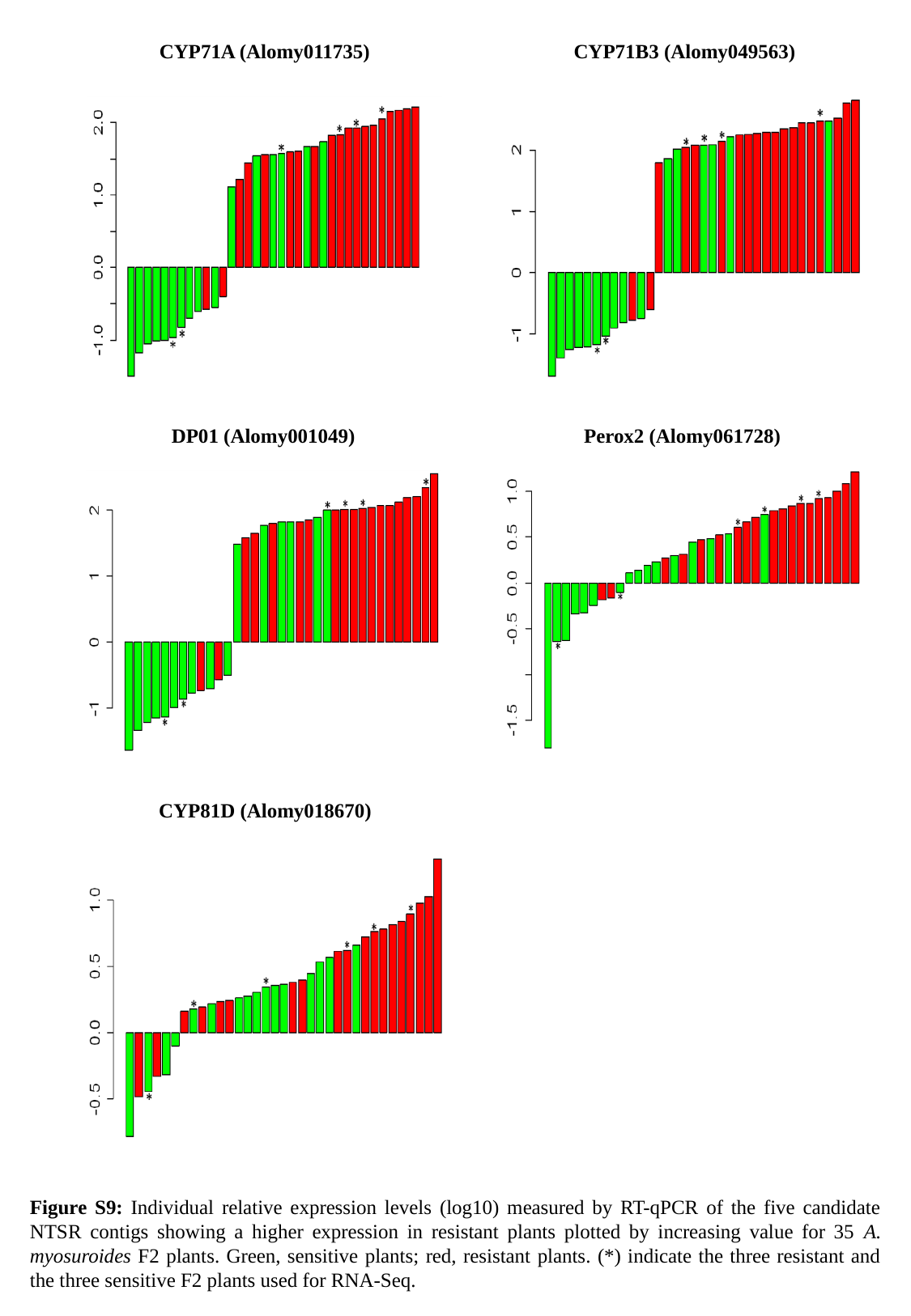

CYP71A (Alomy011735)
CYP71B3 (Alomy049563)
DP01 (Alomy001049)
Perox2 (Alomy061728)
CYP81D (Alomy018670)
Figure S9: Individual relative expression levels (log10) measured by RT-qPCR of the five candidate NTSR contigs showing a higher expression in resistant plants plotted by increasing value for 35 A. myosuroides F2 plants. Green, sensitive plants; red, resistant plants. (*) indicate the three resistant and the three sensitive F2 plants used for RNA-Seq.
